# Identification and Characterization of Nucleolin as a COUP-TFII Coactivator of Retinoic Acid Receptor β Transcription in Breast Cancer Cells

**DOI:** 10.1371/journal.pone.0038278

**Published:** 2012-05-31

**Authors:** Lacey M. Litchfield, Krista A. Riggs, Alyson M. Hockenberry, Laura D. Oliver, Katelyn G. Barnhart, Jian Cai, William M. Pierce, Margarita M. Ivanova, Paula J. Bates, Savitri N. Appana, Susmita Datta, Piotr Kulesza, Jean McBryan, Leonie S. Young, Carolyn M. Klinge

**Affiliations:** 1 Department of Biochemistry & Molecular Biology and Center for Genetics and Molecular Medicine, Louisville, Kentucky, United States of America; 2 Department of Pharmacology and Toxicology, University of Louisville School of Medicine, Louisville, Kentucky, United States of America; 3 James Graham Brown Cancer Center, Louisville, Kentucky, United States of America; 4 Department of Bioinformatics and Biostatistics, University of Louisville School of Public Health and Information Sciences, Louisville, Kentucky, United States of America; 5 Department of Pathology, Feinberg School of Medicine, Northwestern University, Chicago, Illinois, United States of America; 6 Endocrine Oncology Research Group, Department of Surgery, Royal College of Surgeons in Ireland, Dublin, Ireland; II Università di Napoli, Italy

## Abstract

**Introduction:**

The orphan nuclear receptor COUP-TFII plays an undefined role in breast cancer. Previously we reported lower COUP-TFII expression in tamoxifen/endocrine- resistant *versus* sensitive breast cancer cell lines. The identification of COUP-TFII-interacting proteins will help to elucidate its mechanism of action as a transcriptional regulator in breast cancer.

**Results:**

FLAG-affinity purification and multidimensional protein identification technology (MudPIT) identified nucleolin among the proteins interacting with COUP-TFII in MCF-7 tamoxifen-sensitive breast cancer cells. Interaction of COUP-TFII and nucleolin was confirmed by coimmunoprecipitation of endogenous proteins in MCF-7 and T47D breast cancer cells. *In vitro* studies revealed that COUP-TFII interacts with the C-terminal arginine-glycine repeat (RGG) domain of nucleolin. Functional interaction between COUP-TFII and nucleolin was indicated by studies showing that siRNA knockdown of nucleolin and an oligonucleotide aptamer that targets nucleolin, AS1411, inhibited endogenous COUP-TFII-stimulated *RARB2* expression in MCF-7 and T47D cells. Chromatin immunoprecipitation revealed COUP-TFII occupancy of the *RARB2* promoter was increased by all-*trans* retinoic acid (atRA). RARβ2 regulated gene *RRIG1* was increased by atRA and COUP-TFII transfection and inhibited by siCOUP-TFII. Immunohistochemical staining of breast tumor microarrays showed nuclear COUP-TFII and nucleolin staining was correlated in invasive ductal carcinomas. COUP-TFII staining correlated with ERα, SRC-1, AIB1, Pea3, MMP2, and phospho-Src and was reduced with increased tumor grade.

**Conclusions:**

Our data indicate that nucleolin plays a coregulatory role in transcriptional regulation of the tumor suppressor *RARB2* by COUP-TFII.

## Introduction

COUP-TFI (*NR2F1*) and COUP-TFII (*NR2F2*) are ‘orphan’ members of the steroid/nuclear receptor (NR) superfamily [1] COUP-TFs regulate gene transcription in a cell- and gene- specific manner. COUP-TFII differs from COUP-TFI at the N-terminus, but is conserved within the DNA binding and ligand binding domains (DBD and LBD) [1]. Gene knockout mice demonstrated that COUP-TFI (*Nr2f1*) and COUP-TFII (*Nr2f2*) have distinct roles during embryogenesis, notably in the nervous and cardiovascular systems, respectively [2], [3]. Although COUP-TFs are classified as orphan receptors, as they have no currently established physiological ligands, the crystal structure of the COUP-TFII LBD showed that its “auto-repressed conformation” was relieved by 9-*cis* and all-*trans* retinoic acids (9cRA and atRA) that bind the LBD with ∼17–26 µM affinity [4].

While the precise gene changes and epigenetic events that lead to breast tumorigenesis are still under investigation [5], [6], [7], estrogens are well-established risk factors in breast cancer [8]. Adjuvant endocrine therapies including the use of antiestrogens, *e.g.*, tamoxifen (TAM), and aromatase inhibitors (AI), *e.g.*, letrozole, are effective in reducing disease recurrence in many patients [9]. Antiestrogens, including TAM and fulvestrant, work by targeting estrogen receptor α (ERα) because of its proliferative activity in breast tumors [10], [11]. AI work by blocking the synthesis of estrogens from androgenic precursors including androstenedione and testosterone [12].

Altered gene expression can dictate both the formation of tumors and patient response to treatment. In breast cancer, conflicting evidence has been reported on the nature of COUP-TFII in either promoting or inhibiting cancer formation, as well as influencing patient survival with adjuvant therapy. COUP-TFII is not expressed in basal-like, triple negative, dedifferentiated MDA-MB-231 and is lower in tamoxifen (TAM)/endocrine-resistant LCC9 and LY2 breast cancer cells than in parental endocrine-sensitive MCF-7 cells, whereas COUP-TFI expression is similar [13], suggesting a role in maintenance of differentiation and endocrine sensitivity. In agreement with this data, COUP-TFII was reduced in some ERα-null breast cancer cell lines [14]. These results suggest that, like ERα, loss of COUP-TFII may be considered an indicator of poor prognosis. Other reports suggested that COUP-TFII may play a role in mammary tumor formation in mice and that COUP-TFII expression in human breast tumors is associated with reduced survival [15], [16]. These conflicting findings may be resolved through further investigation of the activities of COUP-TFII in breast cancer.

The function of COUP-TFs as transcription factors that can either suppress or stimulate gene transcription is dependent on interactions with other proteins. COUP-TFI [17] and COUP-TFII [18] interact with corepressors NCoR and SMRT. Proteins interacting with COUP-TFI include Sp1 [19]; the viral transactivator Tat [20]; CTIP1 and CTIP2, HDACs 1 and 2, and a nucleosome remodeling and deacetylation (NuRD) complex [21]; ERα [22], [23]; AhR [24]; and many coregulators (reviewed in [25]). Twenty-four proteins interacted with HA-FLAG-COUP-TFI in stably-transfected HeLaS3 cells [26]. Interaction of ORCA with the COUP-TFII LBD stimulated transcriptional activation of the rat hydratase-dehydrogenase gene promoter in transiently transfected Bsc40 monkey kidney cells [27]. COUP-TFII interacted with the hinge domain of the glucocorticoid receptor α (GRα) and repressed phosphoenolpyruvate carboxykinase gene transcription [28]. No one has, to our knowledge, reported proteomic identification of COUP-TFII-interacting proteins.

The focus of the present study was to identify proteins that interact with COUP-TFII in MCF-7 cells to gain new insights into COUP-TFII's role in breast cancer. Nucleolin was identified among the nuclear proteins interacting with COUP-TFII. COUP-TFII-nucleolin interaction was confirmed by co-immunoprecipitation. This study reports a significant inverse association of COUP-TFII with breast tumor grade. Expression of the tumor suppressor retinoic acid receptor β2 (RARβ2), reduced in breast cancer [29], [30], and dependent on COUP-TFII [31] was increased by nucleolin overexpression. Our data indicate that nucleolin plays a coregulatory role in COUP-TFII transcriptional regulation of *RARB2*.

## Materials and Methods

### Chemicals

4-hydroxytamoxifen (4-OHT) and 9-*cis* and all-*trans* retinoic acid (9cRA and atRA) were from Sigma (St. Louis, MO). ICI 182,780 (Fulvestrant) was from Tocris (Ellisville, MO). Sequences of AS1411 (AGRO100, an antiproliferative, 26-mer G-rich oligonucleotide) and an inactive negative control C-rich control oligonucleotide (CRO) were reported [32] and purchased from Integrated DNA Technologies, Inc. (Coralville, IA).

### Antibodies and reagents

The following antibodies were purchased: polyclonal COUP-TFII (Abcam, Cambridge, MA); monoclonal (mAB) anti-human COUP-TFII (R&D systems, Minneapolis, MN; PP-H7147-00. 2ZH7147H); mAB anti-FLAG M2 and β-actin (Sigma); polyclonal nucleolin (NB600-241, Novus Biologicals, Littleton, CO), monoclonal nucleolin/C23 (MS-3; Santa Cruz Biotechnology, Santa Cruz, CA); MBP-probe (R3.2; Santa Cruz Biotechnology); and HDAC-1 (Santa Cruz Biotechnology). HRP–conjugated secondary antibodies were from GE Healthcare (Piscataway, NJ).

Goat anti-rabbit and anti-mouse magnetic beads were from Thermo Scientific (Waltham, MA). *In vitro* transcription/translation used PROTEINScript II kit (Ambion, Austin, TX) or TNT Quick Coupled Transcription/Translation (Promega, Madison, WI).

### Plasmid Construction

Human COUP-TFII cDNA was amplified from DNA from MCF-7 cells using Pfx DNA polymerase (Invitrogen, Carlsbad, CA). The forward primer contained an *Eco*RI restriction site (5′-CCGAATTCGATATGGCAATGGTAGTTAGCACG-3′) and the reverse primer was designed to remove the stop codon from COUP-TFII and add a *Xho*I restriction site (5′-GTCCTCGAGTCGTTGAATTGCCATATACGGCCA-3′). The resulting fragment was cloned into pIRES-GFP-1a (Stratagene, Santa Clara, CA) to construct a C-terminal FLAG-tagged COUP-TFII expression plasmid (pIRES-COUP-TFII-FLAG). The inclusion of COUP-TFII-FLAG in the resulting pIRES-COUP-TFII-FLAG plasmid was verifed by DNA sequencing and western blot analysis (Figure S1).

### Cell culture

MCF-7 and T47D breast cancer cells were purchased from ATCC and used at passage <10. T47D were grown in RPMI 1640 (Invitrogen) supplemented with 5% FBS and 6 µg/ml insulin (Sigma). MCF-7 cells were maintained as described [13].

### Affinity purification and identification of COUP-TFII-FLAG interacting proteins

One mg of whole cell extract (WCE), prepared as described in [13], from MCF-7 cells transfected (24 h) with pIRES-COUP-TFII-FLAG as described in Methods S1 was incubated with EZ view™ Red ANTI-FLAG® M2 Agarose Affinity gel (Sigma) overnight (∼16 h) at 4°C with constant rotation. COUP-TFII-FLAG interacting proteins were eluted using two methods: 1) 0.1 M glycine, pH 3.5, 15 min at room temperature with constant rotation; 2) an additional incubation with 0.1 M glycine, pH 3.5, 5 min at 95°C (Fig. S2A).

### Protein identification by multidimensional protein identification technology (MudPIT)

Proteins eluted from the FLAG-affinity gel were trypsin digested and processed for mass spectrometry as detailed in Methods S1. MS/MS spectra of the peptides were acquired by Q-TOF mass spectrometer (Waters, Milford, MA) in data dependent mode. Proteins were identified by comparing MS/MS spectra with sequences in Swiss-Prot database by ProteinLynx from Waters.

### Co-immunoprecipitation (co-IP) and immunoblotting

Nuclear and cytosolic proteins were harvested in lysis buffer (10 mM HEPES pH 7.9, 1.5 mM MgCl_2_, and 10 mM KCl) containing 0.1 M DTT, protease and phosphate inhibitors (Roche, Indianapolis, IN). Following centrifugation, the supernatant containing cytosolic extract (CE) was collected. The pellet was resuspended in nuclear extraction buffer (20 mM HEPES pH 7.9, 1.5 mM MgCl_2_, 0.4 M NaCl, 0.2 M EDTA, and 25% (v/v) glycerol), 0.1 M DTT, protease and phosphatase inhibitors. Nuclei were disrupted by sonication and the nuclear extracts (NE) were collected after centrifugation.

For IP, 4 µg COUP-TFII polyclonal antibody, nucleolin monoclonal antibody, rabbit or mouse IgG (Abcam, Santa Cruz) were added to 250 µl prewashed MagnaBind goat anti-rabbit or anti-mouse IgG beads in RIPA buffer (Sigma) containing DTT, protease and phosphatase inhibitors for 30 min at 4°C. 200–400 µg NE was added and incubated for 4 h at 4°C. Antibody-bound beads were incubated with buffer without NE as an additional negative control. Beads were washed 2× with RIPA buffer, resuspended in 1× Laemmli loading buffer (BioRad, Hercules, CA), separated by SDS-PAGE and analyzed by western blot [13].

### *In vitro* transcribed-translated COUP-TFII interaction with purified recombinant maltose binding protein (MBP)–nucleolin fusion proteins

Extracts from *E. coli* expressing MBP-tagged nucleolin constructs, a gift from Dr. Nancy Maizels [33], were prepared in column buffer (CB, 20 mM Tris-HCl (pH 7.4), 0.2 mM NaCl, 1 mM EDTA) with 0.1 mM PMSF. 200 µg crude extract was incubated with 100 µL amylose resin (New England Biolabs, Ipswich, MA) for 2 h at 4°C. After washing with CB, 20 µL of *in vitro* transcribed/translated COUP-TFII (pIRES-COUP-TFII-FLAG) was added to the amylose resin for 2 h at 4°C. After washing 3× with CB, bound proteins were eluted with 50 µL of 1× Laemmli loading buffer.

### Immunofluorescence staining of COUP-TFII and nucleolin

MCF-7 cells were grown on culture slides (BD Biosciences, Bedford, MA) and fixed with cold methanol. Cells were permeabilized with 0.2% Triton X-100. After blocking with 10% BSA in PBS for 1 h, primary monoclonal COUP-TFII (R&D) and polyclonal nucleolin (Novus Biologicals) antibodies were added (1∶100) for 2 h. The cells were stained with secondary anti-mouse antibody labeled with DyLight™ 488 or anti-rabbit antibody labeled with rhodamine (TRITC) (Jackson ImmunoResearch, West Grove, PA) (1∶500). Cells were incubated with Hoechst (2,5′-Bi-1H-benzimidazole, Invitrogen) for 10 min. Images were captured using an Olympus FV1000 confocal microscope with a 40× objective lens using FluoView™ software.

### Immunohistochemistry of breast tissue microarrays (TMA)

COUP-TFII and nucleolin immunohistochemistry (IHC) was performed using commercial breast tissue microarrays BR961 and BR963 (U.S. Biomax) or an in-house TMA constructed following ethical approval from St. Vincent's University Hospital Ethics Board with tissue from 332 primary breast patients, following written informed consent, as previously described [34], [35]. Data on the patients included pathological characteristics (tumor size, grade, lymph node status, estrogen receptor status) as well as treatment with radiotherapy, chemotherapy or tamoxifen. Follow-up data, median 7.72 years, was collected on the patients to determine disease free and overall survival. Staining was called by two independent observers using the Allred scoring system [34]. Xenografted MCF7 and HCT116 tumors were used as positive and negative controls, respectively (data not shown). Anti-Nucleolin antibody (Clone 4E2, Abcam) was diluted at 1∶500 with overnight incubation at 4°C for BR961 and BR963. A metastatic melanoma was used as a positive control for nucleolin (data not shown). COUP-TFII and nucleolin staining were expressed as H-score: product of intensity (0 to 3 scale, 0 = no expression, 3 strongest expression) and frequency (fraction positive, 0–100%).

### Statistical Evaluation of IHC

The univariate associations between COUP-TFII and nucleolin H-scores and categorical predictors used the Kruskal-Wallis test [34]. A multiple linear regression model was used to fit with COUP-TFII and nucleolin H-scores against pathology, tumor grade, and TNM, classification. The TNM staging system classifies tumors according to disease progression based on the tumor size (T), regional lymph node involvement (N), and distant metastasis (M). Upon assignment of TNM, tumors can further be designated into a condensed grade/stage (I–IV) based on disease severity [36]. Comparisons in the ERα-positive invasive ductal carcinoma subset and among TNM classification within tumor grades were examined (t-test). Fisher's exact test was used for categorical variables to compare two proportions. Kaplan Meier estimates of survival functions were computed and the Wilcoxon test was used to compare survival curves. Two-sided P values of <0.05 were considered to be statistically significant.

### Transient transfection

MCF-7 and T47D cells were transfected with a constant amount of total plasmid DNA, pcDNA 3.1 (Promega), pCMV-Tag2 (Stratagene), pcDNA 3.1-mCOUP-TFII (kindly provided by Drs. Sophia and Ming-Jer Tsai [37]), pCMV2-nucleolin [38] using Fugene 6 or HD (Roche) for 24 h prior to treatment with 10 µM CRO (negative control), AS1411, or random oligomer (RO, 5′-GTTCAGCAGTCACGATTCAGTCCAGT-3′) for 6 or 24 h, as indicated. Where indicated, cells were co-treated with 1 µM atRA or 9cRA for 6 h. Transient transfection of MCF-7 cells with the *RARB* promoter tk-luciferase reporter (kindly provided by Dr. Richard M. Niles [39]) and pTK-*Renilla* (Promega), for dual luciferase reporter assays, as described [23].

### RNA Isolation, RT-PCR and Quantitative Real-Time-PCR (QRT-PCR)

RNA was extracted from cells using Trizol (Invitrogen) or RNeasy (Qiagen, Valencia, CA). The High Capacity cDNA Reverse Transcription kit (Applied Biosystems) was used to reverse transcribe total RNA. QRT-PCR for *RARB2*, *NCL*, *ESR1*, *ESR2*, *GAPDH*, and 18S, using Taqman primers and probes as Assays-on-Demand, was performed in the ABI PRISM 7900 SDS 2.1 (Applied Biosystems, Carlsbad, CA). COUP-TFII (*NR2F2*) and *RRIG1* [40] mRNA expression was measured by QRT-PCR using the SYBR green method and normalized by GAPDH [13]. Analysis and fold differences were determined using the comparative CT method. Fold change was calculated from the ΔΔCT values with the formula 2^−ΔΔCT^ and data are relative to EtOH-treated and control vector transfected cells.

### siRNA transfection

For nucleolin, cells were transfected for 48 h with 25 nM (MCF-7) or 10 nM (T47D) nucleolin Stealth Select RNAi or Stealth RNAi Negative Control (Invitrogen) using Lipofectamine RNAiMAX (Invitrogen). For COUP-TFII, MCF-7 cells were transfected for 48 h with 100 pmol *NR2F2 Silencer* Select siRNA (Ambion). Following transfection, cells were treated for 6 h with 1 µM atRA.

### Chromatin immunoprecipitation (ChIP)

MCF-7 cells were transfected with pIRES-COUP-TFII-FLAG or empty vector for 24 h and serum starved for 48 h in media containing 5% dextran-coated charcoal stripped FBS (DCC-FBS) (Atlanta Biologicals, Lawrenceville, GA). Cells were treated with 1 µM atRA or EtOH for 1 h before crosslinking with 1% formaldehyde for 5 min. ChIP was performed using MAGnify ChIP (Invitrogen). Lysates were incubated with anti-FLAG M2 antibody (Sigma) or mouse IgG (Invitrogen). The following primers were used for PCR to amplify the region of the *RARB2* promoter containing a COUP-TFII binding site [31]: F 5′-CAGGGCTGCTGGGAGTTTTTA-3′ and R 5′-GGCATCCCAGTCCTCAAACAGC-3′. Quantitation was performed as described in [41].

### Statistical analysis

Values are reported as ± SEM. Student's *t* test was used for comparisons between control and treatment. One way ANOVA was used for multiple comparisons followed by Student-Newman-Keuls or Dunnett's post-hoc tests using GraphPad Prism. *P* values considered statistically significant are indicated.

## Results

### Identification of COUP-TFII-associated proteins

C-terminal FLAG-tagged COUP-TFII was overexpressed in MCF-7 cells (∼2-fold higher expression compared to COUP-TFII endogenous expression, Fig. S1 and S2) and interacting proteins were captured by immunoprecipitation (IP) with anti-FLAG-affinity gel (Fig. S2A). The negative control was MCF-7 cells transfected with the pIRES-GFP-1a parental vector and parallel purification of nonspecific interacting proteins (Table S1). The capture of COUP-TFII-FLAG by the anti-FLAG affinity gel was demonstrated (Fig. S2C). Serial glycine steps eluted COUP-TFII-FLAG-associated proteins (Fig. S3). In the first elution, 18 proteins having a ‘moderate association’ with COUP-TFII including hsp70, an established NR chaperone that interacts with COUP-TFI [26], were identified (Table S2). The second elution identified 36 more ‘strongly associated’ nuclear proteins, *i.e.*, ribnucleoproteins, histones, DNA repair proteins, and RNA binding proteins, and nucleolin (Table 1), reflecting COUP-TFII nuclear localization.

**Table 1:** Identification of COUP-TFII interacting proteins in MCF-7 cells.

Protein name	Accession	Mass	pl	Matched	Coverage
	(GI)	(Mr)		(No)	(%)
**Histones**					
Histone H1.0	P07305	20719	11.2	1	6.7
Histone H2A type 2-C	Q16777	5919	11.8	1	16.1
Histone H1.4	P10412	21721	11.4	7	4.1
**Proteins involved in cell cycle & proliferation**					
Antigen KI-67	P46013	358525	9.8	6	0.5
Regulator of chromosome condensation	P18754	44941	7.7	4	3.1
Poly [ADP-ribose] polymerase 1 (PARP-1)	P09874	112881	9.2	7	5.7
**Proteins involved in transcription**					
Activated RNA polymerase II transcriptional****coactivator p15	P53999	14255	9.9	1	8.7
**DNA Repair Proteins**					
DNA topoisomerase 1	P11387	90669	9.6	5	1.6
DNA topoisomerase 2-beta (TopoIIβ)	Q02880	183152	8.4	4	2.0
ATP-dependent DNA helicase 2 subunit 1	P12956	69668	6.6	3	2.1
ATP-dependent DNA helicase 2 subunit 2	P13010	82521	5.7	3	3.0
Peptidyl-prolyl cis-trans isomerase B	P23284	22728	9.6	3	10.6
**RNA binding proteins**					
RNA-binding protein FUS	P35637	53394	9.5	3	2.7
Splicing factor, arginine/serine-rich 1	Q07955	27597	10.5	4	12.6
Splicing factor, arginine/serine-rich 9	Q13242	25526	8.9	2	5.4
Splicing factor, arginine/serine-rich 7	Q16629	27350	11.8	2	3.8
**Other nuclear proteins**					
Nonhistone chromosomal protein HMG 14	P05114	10522	10.0	2	24.2
Nucleolin Protein C23	P19338	76167	4.6	3	1.7
Heat shock cognate 71 kDa protein	P11142	70854	5.4	2	4.3
GTP-binding nuclear protein Ran	P62826	24408	7.6	2	9.7
Heat shock 70 kDa protein 1 (hsp70, *HSPA1*)	P08107	70009	5.6	2	4.4

**Figure 1:**
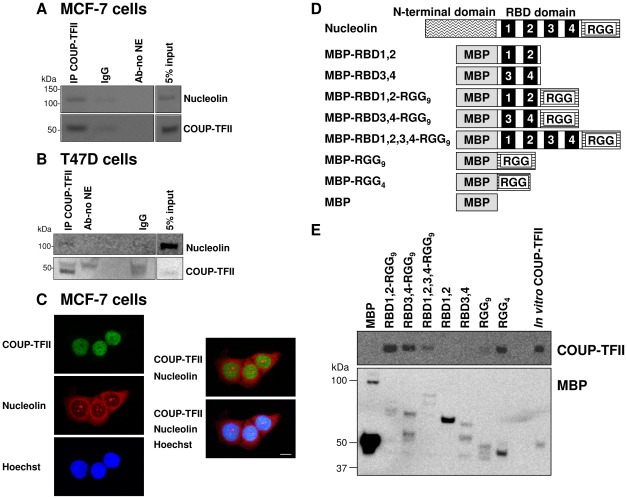
Endogenous nuclear nucleolin-COUP-TFII interaction in MCF-7 and T47D cells. NE (200 µg protein) from MCF-7 cells (A) and (400 µg protein) from T47D (B) cells were immunoprecipitated with COUP-TFII antibody or with rabbit IgG (negative control), followed by western blot analysis for nucleolin and COUP-TFII. 5% input NE serves as loading control. C, Immunofluorescent staining of endogenous COUP-TFII (green) and nucleolin (red) in the nuclei (Hoechst, blue) of MCF-7 cells. Merged images are shown at the right. Bar is 10 µm. D, schematic representation of the N- terminal maltose binding protein (MBP)-tagged recombinant nucleolin proteins used for MBP pull-down assays. MBP was fused to the N-termini of the RNA binding domains (RBD) and/or the arginine/glycine-rich domain (RGG) of nucleolin. E, *In vitro* transcribed/translated COUP-TFII was incubated with the MBP-nucleolin fragments or MBP. Interacting proteins were captured with amylose resin. Eluted proteins were probed for COUP-TFII (top) and MBP (bottom, control).

WCE from pCOUP-TFII-FLAG-transfected MCF-7 cells (Fig. 1) was incubated with anti-FLAG affinity gel, eluted with 0.1 M glycine, pH 3.5 for 5 min. at 95°C, and subjected to MudPIT peptide identification. Matched (No) indicates the number of sequenced peptides that match the full length protein. Coverage indicates the % of the total protein matched. (Only matches of >1 peptide match and/or >3% coverage are included.)

Nucleolin is a multifunctional protein with roles in processes including transcription, ribosome biogenesis, DNA replication, histone chaperone/chromatin remodeling, apoptosis, and macropinocytosis [42], [43], [44], [45]. There are several examples of nucleolin functioning as a transcription factor or as a coregulator through its interactions with other proteins [46], [47], [48]. Because nucleolin plays multiple nuclear roles and is a target of anticancer therapy [43], we selected nucleolin for follow-up studies.

### Endogenous COUP-TFII and nucleolin interact in MCF-7 and T47D cells

We next examined endogenous nucleolin-COUP-TFII interaction in untreated MCF-7 cells. IP with a COUP-TFII antibody detected nucleolin interaction in the NE (Fig. 1A), although nucleolin is in CE as well (Fig. S4A). We did not detect COUP-TFII-nucleolin interaction in CE because COUP-TFII is not in the CE (Fig S4A). Reciprocal co-IP of COUP-TFII with nuclear nucleolin was detected (Fig. S4A). Another example of a COUP-TFII- nucleolin co-IP in MCF-7 cells is also provided (Fig. S4C and D). To examine whether COUP-TFII interacts with nucleolin in another luminal breast cancer cell line, we performed IP with a COUP-TFII antibody in T47D cells and confirmed nucleolin-COUP-TFII interaction in the NE (Fig 1B). Immunofluorescence microscopy revealed a pattern of co-localization of endogenous nucleolin and COUP-TFII in the nucleus, but not within the nucleolus nor in the cytoplasm, of MCF-7 cells (Fig. 1C). These data confirm endogenous COUP-TFII-nucleolin nuclear interaction. Because the focus of this study is COUP-TFII-interacting proteins, we did not evaluate COUP-TFI-nucleolin interaction.

### Direct interaction of COUP-TFII with the RGG domain of nucleolin

To determine if COUP-TFII interacts directly with nucleolin and which domain(s) are involved, *in vitro* transcribed/translated COUP-TFII and MBP-tagged recombinant nucleolin polypeptides were incubated with an amylose affinity resin (Fig. 1D and E). The MBP fusion proteins contain the RNA binding domains (RBD1,2,3,4) and/or the arginine-glycine repeat (RGG). Nine RGG repeats are present in the C-terminus of nucleolin. Only these domains were investigated because the N-terminal domain of nucleolin cannot be expressed in *E. coli* [38]. COUP-TFII was bound to all MBP-tagged polypeptides containing the RGG domain but not with MBP-RBD1,2, MBP-RBD3,4, or MBP alone. RGG9 appears to interact with COUP-TFII with weaker affinity compared to RGG4, perhaps because of the lower abundance of the MBP-RGG9 protein relative to MBP-RGG4. Overall, these results indicate that the C-terminal RGG domain is the minimal domain required for COUP-TFII-nucleolin interaction.

### Immunohistochemical COUP-TFII and nucleolin staining in human breast tissue and tumor tissue microarrays

**Figure 2:**
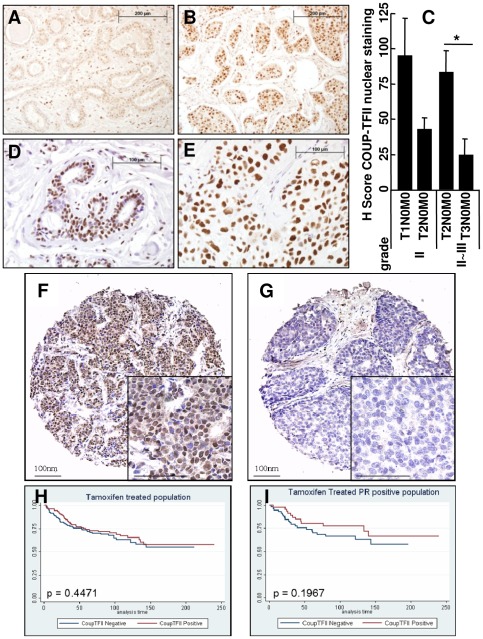
COUP-TFII and nucleolin in breast cancer tissue microarrays. A and B, COUP-TFII immunostaining at 200×: A, benign breast tissue (H-score 30) and B, invasive ductal carcinomas, grade 2 (H-score 153). Bar is 200 mm. C, Average ± SEM of H-score for nuclear COUP-TFII staining in ERα-positive invasive ductal carcinomas by tumor grade. * significantly different from T2N0M0 (p<0.05). D and E, Nucleolin immunostaining at 400×: D, benign breast tissue (H-score 13) and E, invasive ductal carcinomas, grade 3 (H-score 151). Bar is 100 µm. F and G, immunohistochemical localization of COUP-TFII (100×, inset: 200×) on a tissue microarray constructed from archival tissue from 332 breast cancer patients [34] showing positively (F) and negatively (G) stained cores COUP-TFII at 200×, bar is 100 µm. H and I, Kaplan-Meier estimates of disease-free survival functions were computed, and the Wilcoxon test was used to compare survival curves. In addition, the Wilcoxon rank sum test was used to compare two medians. The data are not statistically significant.

Nuclear COUP-TFII and nucleolin immunoreactivity were examined in two independent human breast tissue microarrays (TMAs, Fig. 2). In the TMAs from U.S. Biomax, significant differences in COUP-TFII staining were observed between TNM classes of tumor grades II (d = −51.7, p = 0.078) and II∼III (d = −58.5, p = 0.046) (Fig. 2C). COUP-TFII and nucleolin staining were correlated in invasive ductal carcinomas (ϕ_p_ = 0.31, p = 0.0281; ϕ_s_ = 0.30, p = 0.0334). Normal breast tissue was also positive for COUP-TFII expression (Average H-score 127.5, SEM 5; data not shown). In a separate breast TMA [34], staining of a total of 332 patient tumors showed ∼47% were positive for nuclear COUP-TFII (Table 2, Fig. 2F). There was a significant association between tumor grade and COUP-TFII with high grade tumors tending to be COUP-TFII negative (Table 2). COUP-TFII was also significantly positively associated with ERα, SRC-1, PEA3, MMP2, and phospho-Src and negatively associated with HER2 (Table 2). High SRC-1 (*NCOA1*) was associated with a favorable response to TAM [49], a finding that corresponds to COUP-TFII's role in 4-OHT-medicated inhibition of breast cancer cell proliferation [13]. However, breast tumors from aromatase inhibitor-resistant patients show high expression of SRC-1 and a reduction in disease-free survival [50]; thus, the relationship between COUP-TFII and SRC-1 expression will require further investigation. SRC-1 and PEA3 synergistically activated COUP-TFII-promoter luciferase activity in transiently transfected HeLa cells [51]. PEA3 directly activates MMP2 transcription [35]; hence COUP-TFII may be correlated with MMP2 through the PEA3-COUP-TFII connection, although this hypothesis will require further analysis beyond the scope of the present study. The relationship between COUP-TFII and phospho-Src may be because activation of Src is part of the MAPK pathway that increases COUP-TFII expression [52]. According to Kaplan Meier, COUP-TFII did not associate with disease free survival in tamoxifen-treated patients (p = 0.4471, Fig. 2H and I).

**Table 2:** COUP-TFII staining in breast tumor microarray.

	Total Population	% COUPTFII positive	p value
	n = 321	47%	
**ER**			
neg	99	34.3	
pos	222	52.3	**0.004**
**PR**			
neg	133	50.4	
pos	146	44.5	0.339
**Her2**			
neg	267	50.2	
pos	62	32.3	**0.011**
**Grade**			
1	24	54.2	
2	122	54.1	
3	112	33.0	**0.003**
**Node**			
neg	156	47.4	
pos	167	46.1	0.824
**Recurrence**			
neg	200	46.0	
pos	132	47.7	0.822
**SRC-1**			
neg	214	41.6	
pos	113	56.6	**0.011**
**AIB1**			
neg	88	25.0	
pos	221	55.7	**<.001**
**Pea3**			
neg	120	35.8	
pos	120	58.3	**0.001**
**MMP2**			
neg	29	20.7	
pos	284	48.2	**0.005**
**psrc**			
neg	226	42.0	
pos	87	56.3	**0.031**

Associated expression of COUP-TFII with ERα, PR, HER2, SRC-1, AIB1, Pea3, AIB1, MMP2, and phospho-Src (psrc) staining in 560 human breast tumors [34], [35]. Statistical analysis was performed using the Fisher's exact test, and a P value of <0.05 is considered to be significant (**bold** values).

### atRA enhances COUP-TFII binding to the *RARB2* promoter

**Figure 3:**
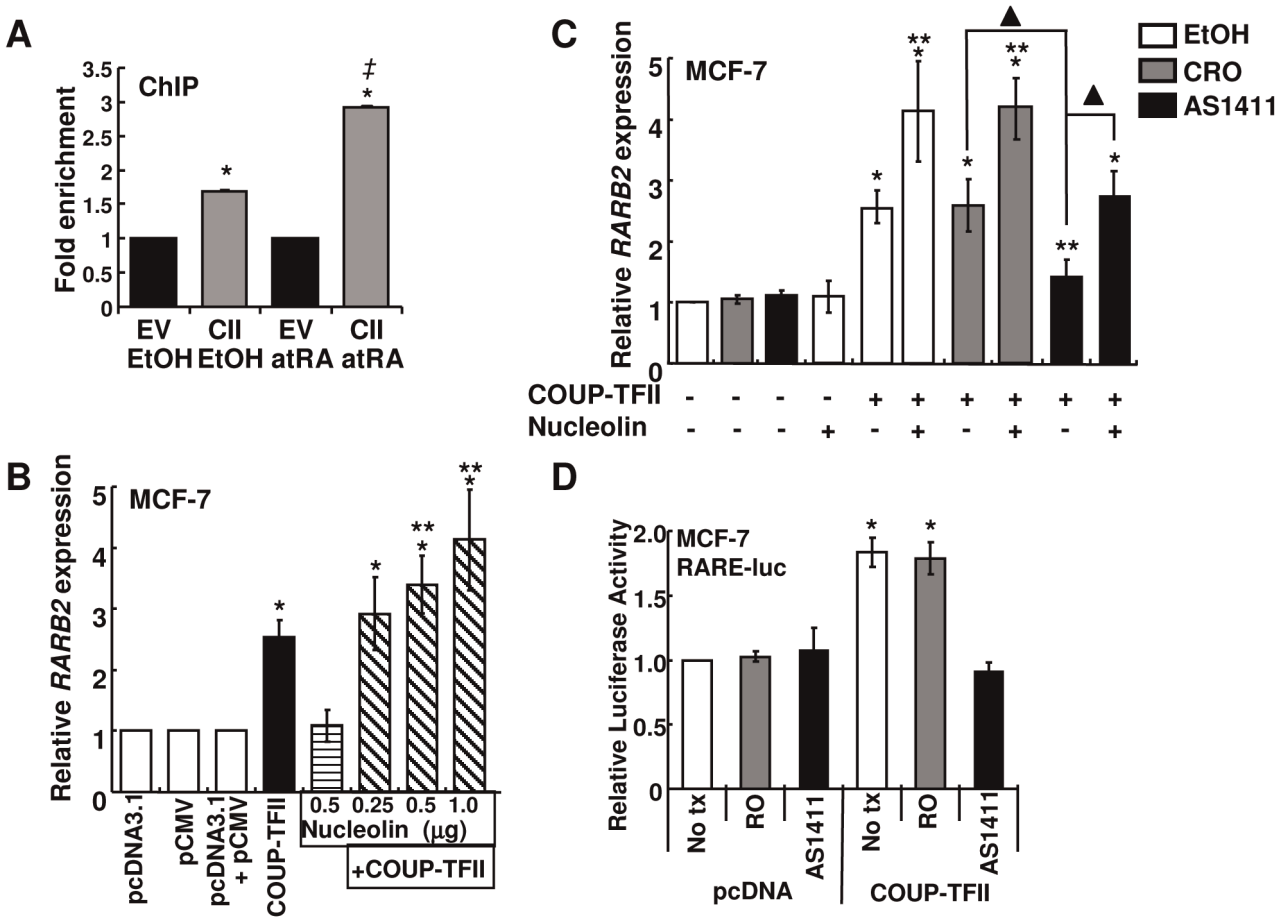
COUP-TFII increases *RARβ2* transcription in MCF-7 cells. A, ChIP of COUP-TFII-FLAG to the *RARB2* promoter in MCF-7 cells transfected with empty vector (EV) or COUP-TFII (CII) and treated with EtOH or 1 µM atRA for 6 h. * P<0.05 *versus* E control, *‡* P<0.05 *versus* CII-EtOH. B-C, Cells were transfected with parental or expression plasmids for COUP-TFII or nucleolin for 24 h and were treated with EtOH, 10 µM CRO or AS1411 for 24 h post-transfection. For C, cells were transfected with 0.5 µg and 1 µg of nucleolin and COUP-TFII expression vector, respectively. Q-PCR was performed to determine *RARB2* expression. Values are the average of 6 separate experiments ± SEM. D, Cells were transfected with pcDNA or pcCOUP-TFII and treated with 10 µM RO or AS1411 for 24 h. Dual luciferase activity was expressed relative to the pcDNA-transfected, no-treatment control. Values are mean ± S.E.M. of two separate experiments. For B–D, * P<0.05 *versus* vector control, ** COUP-TFII alone, or ▴ between the indicated values.

COUP-TFII is required for atRA- or 9cRA- induced *RARB2* expression in breast cancer cells [31], [53] and binds the *RARB2* promoter in electrophoretic mobility shift assays [31]. To examine COUP-TFII interaction with the *RARB2* promoter, we first examined recruitment of endogenous COUP-TFII to the *RARB2* promoter using the R&D systems COUP-TFII antibody, but were unable to detect product in the PCR reactions (data not shown) likely due to lower COUP-TFII protein in MCF-7 cells compared to C3H10T1/2 CH3 mouse embryo mesenchymal cells that express high levels of COUP-TFII [54]. To obviate this difficulty, MCF-7 cells were transfected with an empty vector or COUP-TFII-FLAG and ChIP was performed after FLAG IP. ChIP revealed for the first time that COUP-TFII binds the *RARB2* promoter and atRA increased COUP-TFII occupancy at the *RARB2* promoter by 72% in MCF-7 cells (Fig. 3A, Fig. S5).

### AS1411 inhibits COUP-TFII-stimulated *RARB2* gene expression in MCF-7 and T47D breast cancer cells

Once establishing the presence of COUP-TFII at the promoter of its target gene *RARB2*, we sought to determine if nucleolin functions as a coactivator for COUP-TFII-mediated *RARB2* expression. *RARB2* was increased in MCF-7 cells transfected with COUP-TFII and nucleolin overexpression potentiated the *RARB2* induction in a concentration-dependent manner (Fig. 3B). Further, because the G-rich, G-quartet forming DNA aptamer AS1411 binds and reduces nucleolin activity [43], [55], we hypothesized that AS1411 would inhibit COUP-TFII-stimulated *RARB2* expression. AS1411 inhibited the COUP-TFII-induced increase in *RARB2*, while CRO (negative control) had no effect (Fig. 3C). Although nucleolin did not affect basal *RARB2* expression, nucleolin abrogated the inhibition of *RARB2* transcription by AS1411 (Fig. 3C). COUP-TFII also increased luciferase activity from a *RARB* gene promoter-reporter and AS1411 abrogated luciferase induction (Fig. 3D).

**Figure 4:**
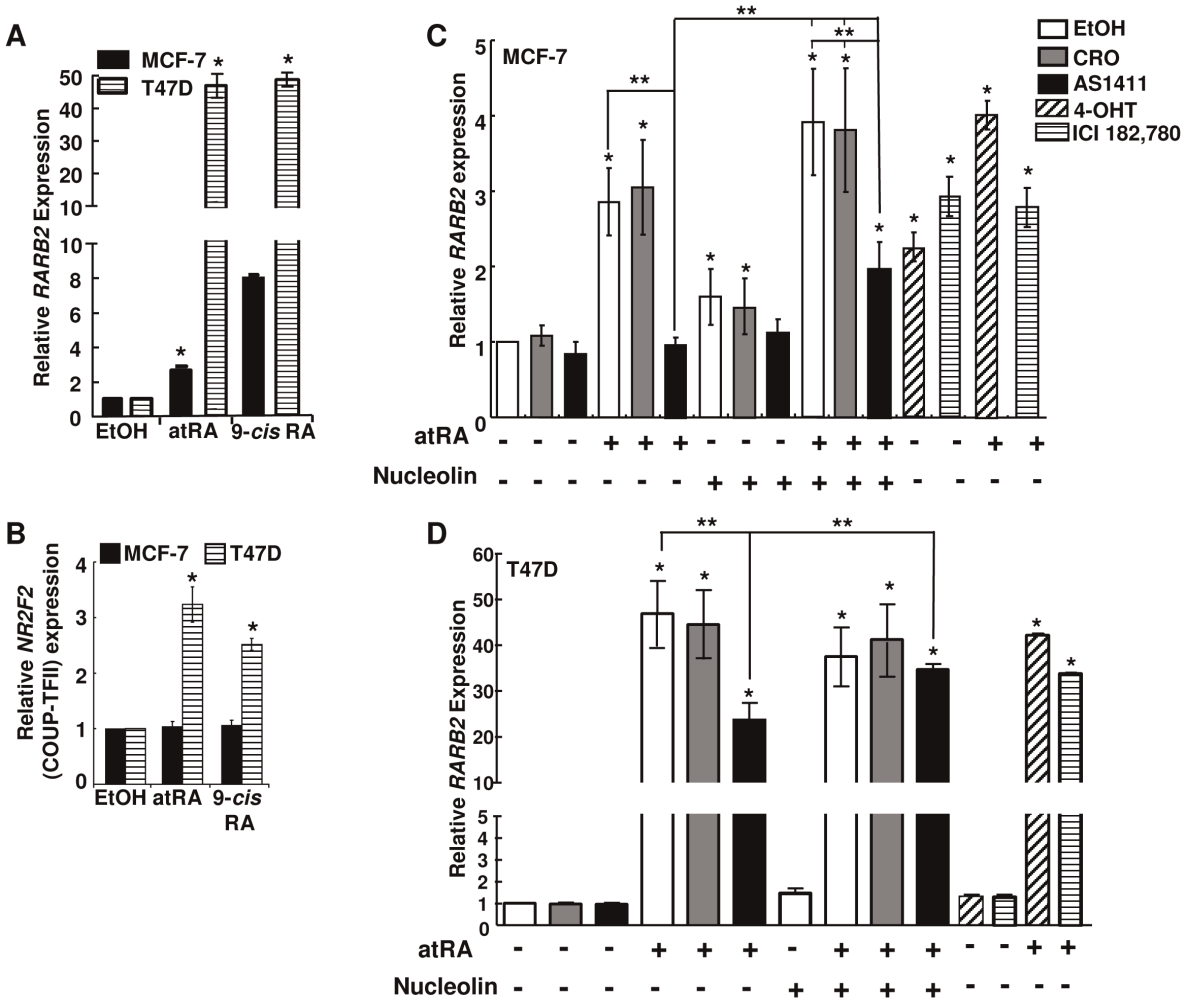
Regulation of *RARβ2* transcription. A and B, Q-PCR for *RARB2* (B RARβ2) and *NR2F2* (C COUP-TFII) in MCF-7 or T47D cells treated with EtOH, 1 µM atRA, or 1 µM 9-cis-RA for 24 h. Values are the average of 3–5 separate experiments. * P<0.05 *versus* EtOH. C, MCF-7 and D, T47D cells were transfected with 2 µg pCMV-tag2 (−) or pCMV-tag2-nucleolin (+) for 24 h prior to 24 h treatment with EtOH, 10 µM CRO, or 10 µM AS1411. Where indicated, cells were treated with 1 µM atRA, 100 nM 4-OHT, or 100 nM ICI 182,780 for 6 h. Q-PCR for *RARB2* expression. Values are the average of 6 (MCF-7) and 4–10 (T47D) separate experiments ± SEM. * P<0.05 *versus* EtOH or ** between the indicated values.

To be sure that any effect of AS1411 on *RARB2* is not cell-line specific, MCF-7 and T47D cells with similar nucleolin expression (Fig. S6) were tested. T47D has ∼40% lower COUP-TFII than MCF-7 (Fig. S6). Both atRA and 9cRA increased *RARB2* expression in MCF-7 and T47D cells, with greater induction in T47D, and increased *NR2F2* (COUP-TFII) expression in T47D (Fig. 4A–B).

Pretreatment of MCF-7 cells with AS1411, but not negative control CRO, reduced atRA-induced *RARB2* expression (Fig. 4C). Transfection of MCF-7 cells with a nucleolin expression vector increased basal *RARB2* except in AS1411-treated cells. Nucleolin and atRA additively increased *RARB2* expression in MCF-7 cells and AS1411, but not CRO, reduced *RARB2* induction. Nucleolin significantly abrogated the inhibition of *RARB2* expression by AS1411. COUP-TFII mRNA levels were not significantly reduced by AS1411 (Fig. S7). Neither ER antagonists ICI 182,780 nor 4-OHT blocked atRA-induced *RARB2* expression, indicating that ER is not involved in COUP-TFII-activated *RARB2* expression (Fig. 4C). ICI and 4-OHT increased basal *RARB2* in MCF-7, in agreement with *RARB*-luciferase reporter activation in transfected MCF-7 cells [56].

Pretreatment of T47D cells with AS1411, but not CRO, reduced atRA-induced *RARB2* expression and co-transfection with an expression vector for nucleolin significantly abrogated the inhibition of *RARB2* expression by AS1411 (Fig.4D). AS1411 had no effect on MCF-7 cell viability for the treatment times used in these experiments (Fig. S8), commensurate with previous findings that MCF-7 viability is inhibited only after 6 d of AS1411 treatment [55].

### *RARB2* expression is inhibited by nucleolin or COUP-TFII knockdown

**Figure 5:**
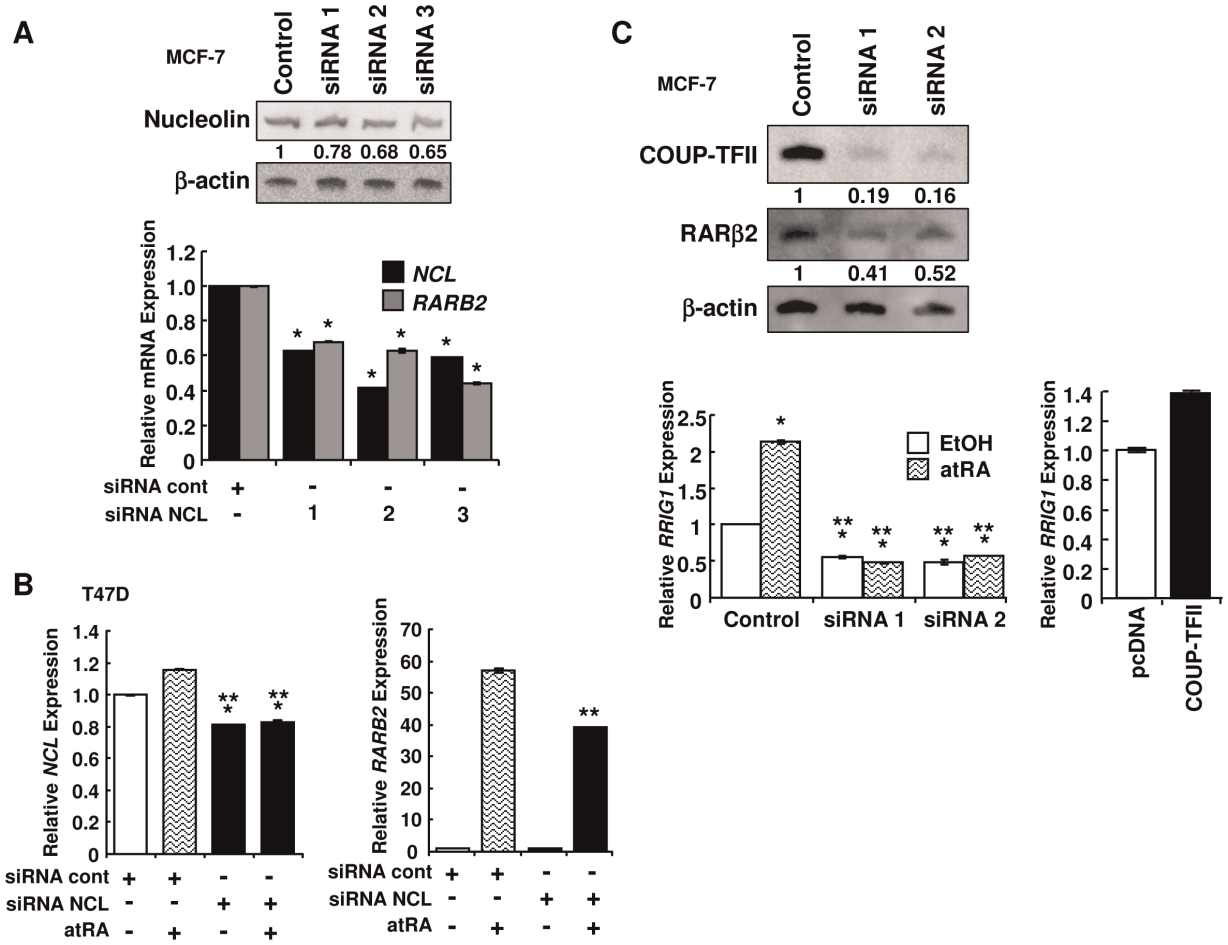
Reduction of COUP-TFII or nucleolin decreases *RARβ2* transcription in MCF-7 cells. MCF-7 (A) and T47D (B) cells were transfected with control siRNA or an siRNA targeting nucleolin for 48 h. T47D cells were treated with EtOH or 1 µM atRA for 24 h. Q-PCR for nucleolin (*NCL*) and *RARB2*. Values are the average of triplicates. C, Western blot showing COUP-TFII and RARβ2 expression after transfection with siCOUP-TFII. Values are relative to β-actin. MCF-7 were transfected with siControl or siCOUP-TFII for 48 h and treated with 1 µM atRA for 6 h. Q-PCR was also performed for *RRIG1*. P<0.001 * *versus* control or ** *versus* atRA.

siNucleolin reduced nucleolin (*NCL*) mRNA by 37–58% and protein by 22–35% (Fig. 5A) in MCF-7 cells. In parallel, basal *RARB2* was reduced 32–56% (Fig. 5A). To determine if nucleolin knockdown inhibited atRA-induced *RARB2*, T47D cells were transfected with siNucleolin and untreated or treated with atRA (Fig. 5B). atRA had no effect on nucleolin knockdown (Fig. 5B). Analogous to the MCF-7 cells, siNucleolin reduced *RARB2* mRNA (Fig. 5B). siNucleolin inhibited the atRA induction of *RARB2* ∼36%. Taken together, results from AS1411 and siNucleolin studies indicate a functional role for nucleolin as a coactivator of COUP-TFII-regulated atRA-induced *RARB2* expression in T47D and MCF-7 cells.

siCOUP-TFII reduced COUP-TFII protein and, consequently, *RARB2* transcription and protein (Fig. 5C). RARβ2 stimulates retinoic acid receptor-induced gene 1 (*RRIG1*) transcription [57]. siCOUP-TFII reduced basal and atRA-induced *RRIG1* expression. Transfection with COUP-TFII increased *RRIG1*. These data substantiate COUP-TFII's regulation of functional RARβ2.

### Effects of AS1411 on nucleolin-COUP-TFII nuclear interaction

**Figure 6:**
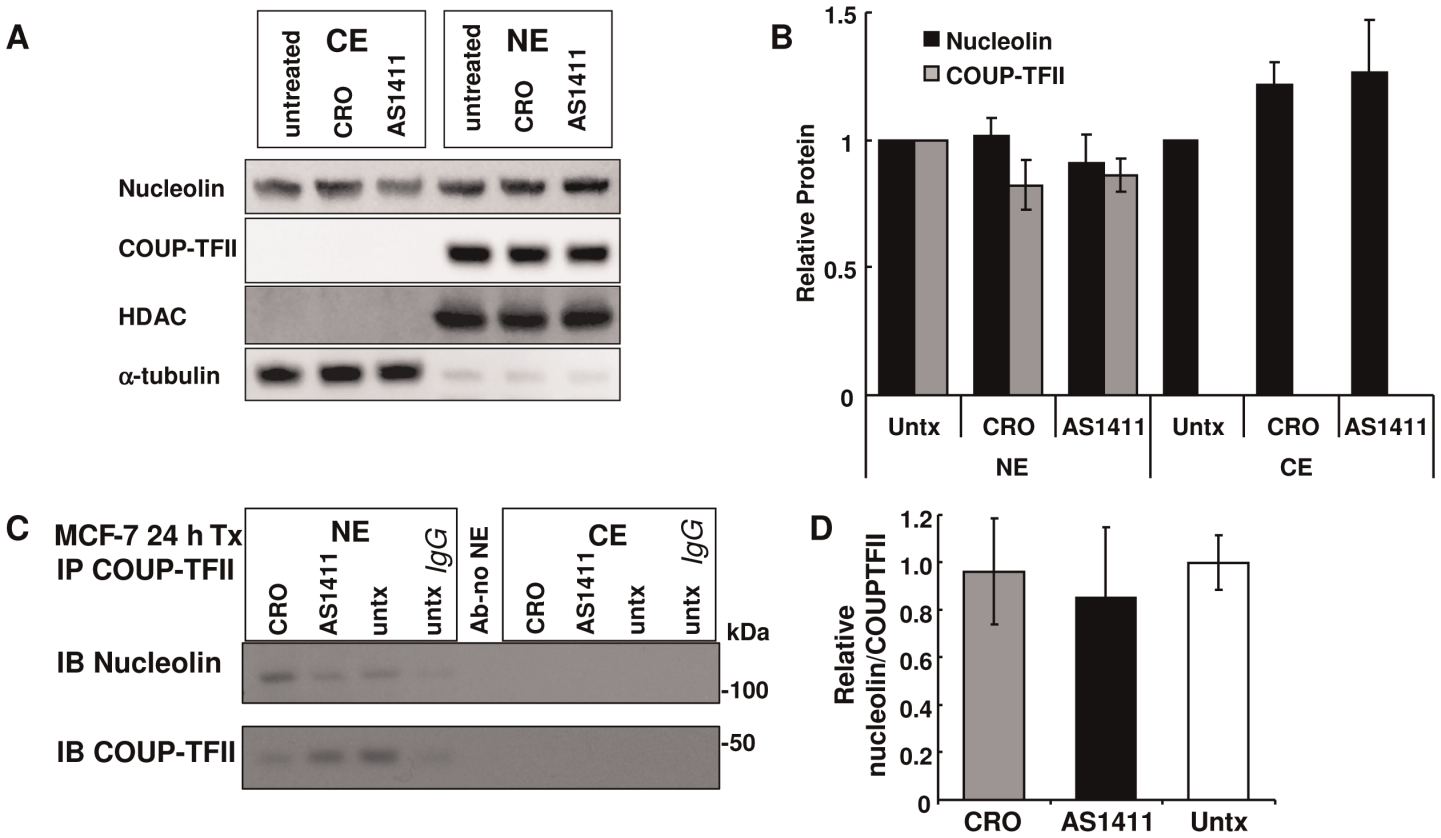
Effects of AS1411 on nuclear nucleolin-COUP-TFII interaction. A, Representative western blots of CE and NE (30 µg) from MCF-7 cells: untreated (untx) or treated with 10 µM CRO or AS1411 for 24 h were probed for nucleolin or COUP-TFII, then stripped and reprobed for HDAC1 and α-tubulin. B, Relative nucleolin and COUP-TFII expression (normalized to respective loading controls and untreated sample protein ratios were set to 1 for NE and CE). Bars are the mean ± SEM of 4–8 separate experiments. C, NE or CE (200 µg) from MCF-7 cells treated as above were IPed with COUP-TFII antibody or rabbit IgG. The Ab-no NE lane was a negative control: COUP-TFII antibody incubated with beads and buffer without NE. D, The ratio of nuclear nucleolin/COUP-TFII is the mean ± SEM of 3 experiments.

AS1411 reduced the nuclear/cytoplasmic ratio of the nucleolin-interacting protein PRMT5 (Protein Arginine Methyltransferase 5) in DU145 prostate cancer cells [38]. AS1411 or CRO did not change nuclear COUP-TFII or nucleolin or cytosolic nucleolin in MCF-7 cells (Fig. 6A–B). There was no change in relative COUP-TFII-nucleolin interaction in MCF-7 cells treated with AS1411 (Fig. 6C–D).

## Discussion

COUP-TFII plays an undefined role in breast cancer [13]. In this report, we identify nucleolin as a new functional partner for COUP-TFII. We have demonstrated for the first time that COUP-TFII binds nucleolin *in vitro* and *in vivo*. In breast tumors, nuclear nucleolin correlates with ERα and cell proliferation [58], [59]. Here IHC staining revealed a correlation between nuclear COUP-TFII and nucleolin staining in invasive ductal carcinoma, a finding that reflects a previous report showing overexpression of COUP-TFII in A549 lung adenocarcinoma cells increased *in vitro* tumorigenicity and migration [60]. We demonstrate that nucleolin acts as a coactivator of endogenous COUP-TFII transcriptional activity for *RARB2* in breast cancer cells. Similarly, nucleolin acted as a coregulator by interacting with c-Jun and Sp1 and increasing cytosolic phospholipase A2 (*cPLA2*) gene transcription [47].

We report a positive correlation between COUP-TFII and ERα, SRC-1, Pea3, MMP2, and phospho-Src expression, an inverse correlation of COUP-TFII with tumor grade and reduced COUP-TFII in ERα-positive, invasive ductal carcinomas with increased TNM stage within tumor grades II and III and HER2 positivity. These data are in agreement with Oncomine analysis demonstrating *NR2F2* is higher in ERα+ tumors and lower in metastatic breast tumors in microarray data [61] (Fig. S9). These observations indicate that COUP TFII may play a role in “phenotype maintenance” and that its function may be restricted to the luminal breast cancer subtypes. We speculate that the precise role of COUP-TFII in breast cancer depends on cellular context, which is consistent with the role of other nuclear receptors in breast cancer [62], and remains to be fully elucidated.

Other COUP-TFII-interacting proteins identified here include PARP-1, which also interacts with nucleolin [63], TOPOIIβ, involved in transcriptional activation by NRs [64], [65], and DNA topoisomerase I (TopoI), which localizes to active transcription sites [66]. Reflecting its NR chaperone role and interaction with COUP-TFI [26], Hsp70 interacted with COUP-TFII. Other COUP-TFII-interacting proteins including hnRNP A2/B1, RPS20, RPL15, and RPL21 were also identified as binding with nucleolin to a c-myc G-quadruplex affinity column [67].

Nucleolin is a key target of the anticancer aptamer AS1411, although AS1411 interacts with other proteins, *e.g.*, NEMO to inhibit NFκB activation and PARP-1 [43]. AS1411 has pleiotropic effects on nucleolin, *e.g.*, inhibiting nucleolin binding to the AU-rich element in the 3′ UTR of *BCL2* in MCF-7 cells causing apoptosis [68] and stimulating macropinocytosis [42]. AS1411 is used to functionally inhibit nucleolin [69]. Here, AS1411 and siNucleolin reduced COUP-TFII-induced *RARB2* expression in MCF-7 and T47D cells and cotransfection with nucleolin reduced AS1411-inhibition. AS1411 did not alter nuclear COUP-TFII-nucleolin interaction, indicating that the mechanism for AS1411 inhibition of *RARB2* expression is independent of reducing nuclear COUP-TFII protein, a result different from AS1411 reducing nuclear PRMT5 in DU145 cells [38]. The inhibition of atRA- and COUP-TFII- regulated *RARB2* expression by AS1411 may also be independent of its effect on nucleolin and may indicate a potential adverse ‘side effect’ of AS1411 that may be a concern if this drug is used for breast cancer therapy.

**Figure 7:**
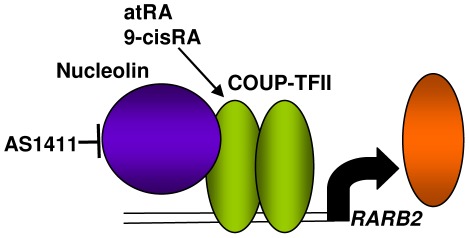
Model of nucleolin-COUP-TFII interaction and upregulation of *RARB2* expression. COUP-TFII binds its response elements as either a homodimer or as a heterodimer with RXR [1]. Previous reports demonstrated that 1) 9cisRA and atRA bind COUP-TFII and increase COUP-TFII transcriptional activity [4]; 2) Nucleolin acts as a transcriptional coregulator by interacting with cJun and Sp1 [47]; 3) COUP-TFI and COUP-TFII increase *RARB2* expression in cooperation with RARα and CBP [31]. Here we demonstrated that 1) nucleolin interacts directly with nuclear COUP-TFII; 2) atRA and 9-cisRA increased *RARB2* mRNA; 3) AS1411, used as a functional inhibitor of nucleolin [43], [69], inhibited COUP-TFII-upregulation of *RARB2* gene transcription; 4) siRNA knockdown of nucleolin reduces induction of *RARB2* and reduced RARβ2 protein.

In conclusion, COUP-TFII interacting proteins were identified in MCF-7 breast cancer cells. Endogenous COUP-TFII and nucleolin interact in both MCF-7 and T47D luminal breast cancer cells. A coregulatory role for nucleolin in COUP-TFII-mediated *RARB2* transcription was described (Fig. 7). Retinoids, *e.g.* 9cRA and atRA, and RARβ have long been associated with tumor suppressive properties such as reduced cell proliferation, inflammation, and solid tumor formation, as well as enhanced apoptosis (reviewed in [70]). RARβ2 expression is reduced in breast tumors and restoration of RARβ2 expression increases sensitivity to tumor growth inhibition by retinoids [71]. Ours is the first demonstration that atRA increased COUP-TFII-*RARβ2* promoter interaction by ChIP. Since serum carotenoids levels are inversely associated with breast cancer risk in women with high mammographic density [72], the increase in *RARB2* in response to COUP-TFII-nucleolin interaction is consistent with a role for COUP-TFII in phenotype maintenance. In agreement with a previous report [31], we observed a much higher induction of *RARB2* in T47D in response to atRA compared to MCF-7 despite, as newly reported here, lower COUP-TFII expression. These data indicate that other mechanisms are also involved. COUP-TFII protein staining in TMAs correlates with ERα expression and an inverse correlation of COUP-TFII with tumor grade in ERα-positive, invasive ductal carcinomas was detected, a finding that correlates with reduced COUP-TFII expression in endocrine-resistant breast cancer cells [13]. Together, these data suggest that COUP-TFII may be important in differentiated ERα-expressing, retinoid-responsive, epithelial breast cancer cells and that reduced COUP-TFII leads to tumor advancement, including endocrine resistance.

## Supporting Information

Figure S1 **Nuclear localization of COUP-TFII in transfected MCF-7 cells.** A, MCF-7 cells were either non-transfected (control) or transfected with pCOUP-TFII-FLAG for 48 h. Immunofluorescence staining was performed for FLAG as described in Methods S1. Cells were counterstained with DAPI (blue) to image nuclei. The bar is 20 µm. Overlap images indicate localization of COUP-TFII-FLAG in the nucleus. B, Western blots of CE (30 µg) or NE (10 µg) from untransfected MCF-7 cells (control) or transfected with pIRES-GFP-1a parental vector, or pCOUP-TFII-FLAG with FLAG or ERα (AER320, ThermoFisher) antibodies. C, Ponceau S staining shows protein levels.
(PDF)

Figure S2 **Overexpression of COUP-TFII-FLAG in MCF-7 cells and immunocapture of COUP-TF-FLAG by the anti-FLAG agarose affinity resin.** A, Briefly, MCF-7 cells were transiently transfected with C-terminal FLAG-tagged COUP-TFII or empty vector for 24 h as described in Materials and Methods. WCEs were prepared and incubated with EZ view™ Red ANTI-FLAG® M2 Affinity gel (Sigma) for 16 h. After rinsing, proteins were eluted with serial glycine elutions: 1) 15 min at room temperature (RT) for proteins associating with immobilized COUP-TFII-FLAG with moderate affinity and 2) 5 min at 95°C to elute proteins bound to the immobilized COUP-TFII-FLAG with high affinity. Immunoprecipitating proteins were analyzed by MudPIT. Non-specific proteins were subtracted from the total interacting proteins to identify proteins specifically interacting with COUP-TFII-FLAG. B, 30 µg of WCE from LCC9, MCF-7 and MCF-7 cells transiently transfected with pCOUP-TFII-FLAG were separated by SDS-PAGE and western blots were performed for COUP-TFII, FLAG and β-actin. Quantitation of the COUP-TFII/β-actin in lanes 2 and 3 indicate a 2-fold increase COUP-TFII in the transfected cells. TAM-R LCC9 cells served as a negative control, as we reported lower COUP-TFII in LCC9 cells compared to parental MCF-7 cells (Riggs *et al* Cancer Res. 66: 10188–98, 2006). Note FLAG signal was only detected in the transfected cells (lane 2), indicating specificity. C, 1 mg of protein in WCE from COUP-TFII-FLAG over-expressing MCF-7 cells were immunocaptured on anti-FLAG agarose affinity beads. COUP-TFII and interacting proteins were eluted with 6 M urea. 30 µg of WCE were separated by SDS-PAGE in parallel to 30 µg unbound IP supernatant and 30 µg eluted protein. COUP-TFII-FLAG affinity bead binding is confirmed by decreased FLAG in the Supernatant (flow-thru = unbound proteins) and enriched FLAG in the eluted samples.
(PDF)

Figure S3 **Testing elution methods for retrieval of COUPTFII-FLAG.** ∼1 mg of WCE from MCF-7 cells transfected with pCOUP-TFII-FLAG was immunocaptured on the anti-FLAG affinity resin and eluted with sequential glycine elutions: 1) 10 or 15 min room temperature (lanes 2 and 4 in Experiments A and B, respectively) or 5 min. at 95°C (lanes 3 and 5) in two different experiments: Exp. A or B, as indicated (lanes 2–5). Samples of the indicated eluates were separated by SDS-PAGE and immunoblotted using anti-FLAG antibody. 20 µg of the IP supernatant, containing unbound COUP-TFII-FLAG, was run in parallel as a control (lane 1). As seen in lane 5, incubation with glycine for 15 min. followed by a 5 min. incubation of fresh glycine at 95°C (Exp. B) eluted the most intact COUP-TFII-FLAG (∼50 kDa). The increased temperature and incubation time of glycine in Exp. B resulted in a FLAG-tagged degradation product(s) of ∼20 kDa (lanes 3–5).
(PDF)

Figure S4 **Endogenous nuclear nucleolin-COUP-TFII interaction in MCF-7 cells.** A, Equal amounts (100 µg) of protein of CE and NE from MCF-7 cells were immunoprecipitated with nucleolin mAB (lanes 3 and 4), mouse (m) IgG (negative control for mAB, lanes 5 and 6), COUP-TFII antisera (lanes 7 and 8), or rabbit (r) IgG (negative control for IPs using COUP-TFII polyclonal antiserum, lanes 9 and 10), followed by western blot for nucleolin and COUPTFII. 20% (20 µg) input NE and CE serve as loading controls (lanes 1 and 2). B, The relative amount of nucleolin and COUP-TFII in the nucleolin IP was plotted relative to expression of each protein in the input (set to 100). COUPTFII in rabbit IgG IPs was not graphed because of the contamination of the heavy IgG chain (lanes 7 and 8, COUP-TFII blot). Western blots demonstrate that: 1) nucleolin interacts with COUP-TFII in the NE of MCF-7 cells (lane 7); 2) nucleolin is not IP'ed with rabbit IgG (lanes 9 and 10); 3) more COUP-TFII interacts with nucleolin in NE IP'ed with nucleolin antibody than with mouse IgG (lane 3 versus lane 5). C, MCF-7 cells were treated with EtOH, 10 nM E2, or 100 nM 4-OHT for 1 h prior to separation of NE and CE. 200 µg of NE or CE were IP'ed with polyclonal COUP-TFII antibody and immunoblotted with a monoclonal antibody (mAB) against nucleolin. The blot was stripped and reprobed with mAB against COUP-TFII. D, 10% input for NE and CE used in IP in part C.
(PDF)

Figure S5 **ChIP of COUP-TFII-FLAG on the** ***RARB2*** **promoter in MCF-7 cells.** A, Chromatin immunoprecipitation was performed in MCF-7 cells transfected with pIRES-COUP-TFII-FLAG or empty vector, serum starved for 48 h, and treated with 1 µM atRA for 1 h. Following Q-PCR using primers to the *RARB2* promoter as described in Materials and Methods, duplicate samples were run on a 2% agarose gel. B, Only EV – EtOH set to 1. atRA increased COUP-TFIIFLAG binding to the RARB2 promoter 32%. Significantly different p<0.05: * to EV – EtOH, ** to EV – atRA, *‡* to CII – EtOH.
(PDF)

Figure S6 **Expression of COUP-TFII and nucleolin in T47D and MCF-7 cells.** A, WCE (50 µg) were Western blotted for COUP-TFII and nucleolin expression. The blot was stripped and re-probed for β-actin. B, The ratio of nucleolin/β-actin and COUP-TFII/β-actin for each cell lines was plotted. These data are the average of 3 separate experiments. The lower COUP-TFII expression in T47D agrees with the higher CT values for NR2F2 in T47D.
(PDF)

Figure S7 **Effect of cell treatments on** ***NR2F2*** **(COUP-TFII) expression in MCF-7 cells.** A, Schematic diagram of transfection and treatment of MCF-7 cells. MCF-7 cells were transfected with equal amounts of pTAG2 control vector or pCMV-nucleolin for 24 h., treated with 10 µM CRO or AS1411, as indicted for 24 h, and 1 µM atRA was added for the last 6 h. RNA was harvested and Q-PCR performed. *NR2F2* (B) values were normalized to GAPDH. Values are the average of 6 separate experiments ± SEM. * significantly different, p<0.05 in one way ANOVA followed by Bonferroni multiple comparison test. Note that there was no statistical difference between the pTAG+AS1411 *versus* Nucl+AS1411 or AS1411 sample measurements of *NR2F2*.
(PDF)

Figure S8 **Neither AS1411 nor CRO inhibit MCF-7 cell viability after 4 d.** MCF-7 cells were treated with the indicated concentrations of AS1411 or CRO and cell viability was measured by an MTT assay (A490 nm, Promega CellTitre assay). Values are the average of 4 determinations ± SEM.
(PDF)

Figure S9 **Oncomine examination of** ***NR2F2*** **expression in breast tumors.** Data mining in Oncomine microarray data sets for *NR2F2* in breast cancer identified that: A, *NR2F2* expression is significantly higher in ERα+ breast tumors (p<0.007). B. *NR2F2* is significantly lower in metastatic breast tumors (p<0.05). Data are from (van de Vijver MJ, He YD, van't Veer LJ, et al. A gene-expression signature as a predictor of survival in breast cancer. N Engl J Med 2002; 347: 1999–2009).
(PDF)

Table S1 **Identification of proteins in MCF-7 WCE that non-specifically (NS) interact with the anti-FLAG-affinity resin.** 5 mg protein in WCE from EMPTY-FLAG vector-transfected MCF-7 cells was incubated with anti-FLAG affinity gel (right side of Supplemental Figure 1), eluted with 0.1 M glycine, pH 3.5 for 15 min. at RT, and subjected to MudPIT peptide identification. Matched number (No) indicates the number of sequenced peptides that match the full length protein. Coverage indicates the % of the total protein matched.
(DOC)

Table S2 **Identification of proteins ‘moderately’ associated with COUP-TFII in MCF-7 cells.** 5 mg protein in WCE from pCOUP-TFII-FLAG transfected, EtOH-treated MCF-7 cells was incubated with anti-FLAG affinity gel (left side of Supplemental Figure 1), eluted with 0.1 M glycine, pH 3.5 for 15 h at RT, and subjected to MudPIT peptide identification. Matched number (No) indicates the number of sequenced peptides that match the full length protein. Coverage indicates the % of the total protein matched. This table excludes proteins that were nonspecifically associated with the anti-FLAG affinity gel as summarized in Supplemental Tables 1.
(DOC)

Methods S1 (DOC)
