# Supplementary material for: Identification and Characterization of Nucleolin as a COUP-TFII Coactivator of Retinoic Acid Receptor β Transcription in Breast Cancer Cells
Source: PLoS One. 2012 May 31;7(5):e38278. doi: 10.1371/journal.pone.0038278 (PMC3365040; doi:10.1371/journal.pone.0038278)
Supplement: Figure S1 — Nuclear localization of COUP-TFII in transfected MCF-7 cells. A, MCF-7 cells were either non-transfected (control) or transfected with pCOUP-TFII-FLAG for 48 h. Immunofluorescence staining was performed for FLAG as described in Methods S1. Cells were counterstained with DAPI (blue) to image nuclei. The bar is 20 µm. Overlap images indicate localization of COUP-TFII-FLAG in the nucleus. B, Western blots of CE (30 µg) or NE (10 µg) from untransfected MCF-7 cells (control) or transfected with pIRES-GFP-1a parental vector, or pCOUP-TFII-FLAG with FLAG or ERα (AER320, ThermoFisher) antibodies. C, Ponceau S staining shows protein levels. (PDF) [file pone.0038278.s001.pdf]

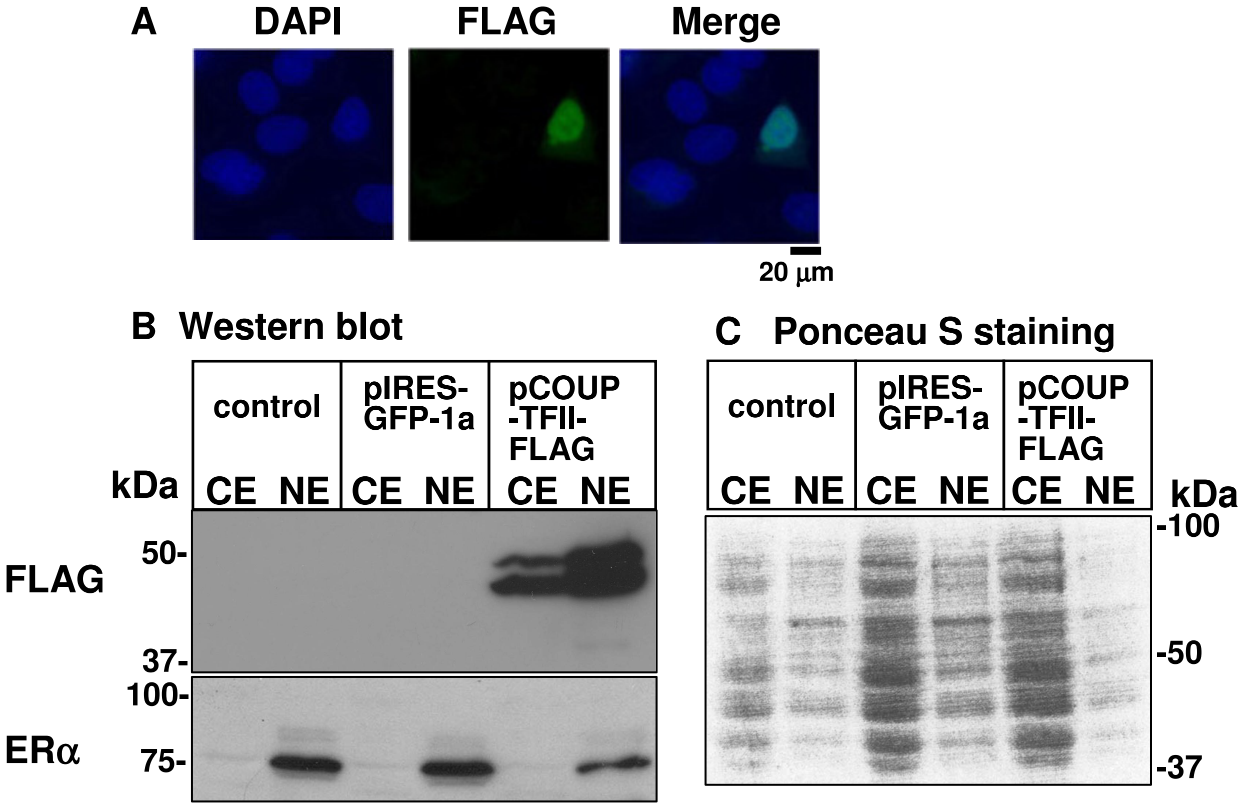

Figure S1: Nuclear localization of COUP-TFII in transfected MCF-7 cells. A, MCF-7 cells were either non-transfected (control) or transfected with pCOUP-TFII-FLAG for 48 h. Immunofluorescence staining was performed for FLAG as described in Supplementary Materials and Methods. Cells were counterstained with DAPI (blue) to image nuclei. The bar is 20 µm. Overlap images indicate localization of COUP-TFII-FLAG in the nucleus. B, Western blots of CE (30 µg) or NE (10 µg) from untransfected MCF-7 cells (control) or transfected with pIRES-GFP-1a parental vector or pCOUP-TFII-FLAG with FLAG or ERα (AER320, ThermoFisher) antibodies. The two bands are likely due to protein degradation. C, Ponceau S staining shows protein levels.
