# Supplementary material for: Identification and Characterization of Nucleolin as a COUP-TFII Coactivator of Retinoic Acid Receptor β Transcription in Breast Cancer Cells
Source: PLoS One. 2012 May 31;7(5):e38278. doi: 10.1371/journal.pone.0038278 (PMC3365040; doi:10.1371/journal.pone.0038278)
Supplement: Figure S2 — Overexpression of COUP-TFII-FLAG in MCF-7 cells and immunocapture of COUP-TF-FLAG by the anti-FLAG agarose affinity resin. A, Briefly, MCF-7 cells were transiently transfected with C-terminal FLAG-tagged COUP-TFII or empty vector for 24 h as described in Materials and Methods. WCEs were prepared and incubated with EZ view™ Red ANTI-FLAG® M2 Affinity gel (Sigma) for 16 h. After rinsing, proteins were eluted with serial glycine elutions: 1) 15 min at room temperature (RT) for proteins associating with immobilized COUP-TFII-FLAG with moderate affinity and 2) 5 min at 95°C to elute proteins bound to the immobilized COUP-TFII-FLAG with high affinity. Immunoprecipitating proteins were analyzed by MudPIT. Non-specific proteins were subtracted from the total interacting proteins to identify proteins specifically interacting with COUP-TFII-FLAG. B, 30 µg of WCE from LCC9, MCF-7 and MCF-7 cells transiently transfected with pCOUP-TFII-FLAG were separated by SDS-PAGE and western blots were performed for COUP-TFII, FLAG and β-actin. Quantitation of the COUP-TFII/β-actin in lanes 2 and 3 indicate a 2-fold increase COUP-TFII in the transfected cells. TAM-R LCC9 cells served as a negative control, as we reported lower COUP-TFII in LCC9 cells compared to parental MCF-7 cells (Riggs et al Cancer Res. 66: 10188–98, 2006). Note FLAG signal was only detected in the transfected cells (lane 2), indicating specificity. C, 1 mg of protein in WCE from COUP-TFII-FLAG over-expressing MCF-7 cells were immunocaptured on anti-FLAG agarose affinity beads. COUP-TFII and interacting proteins were eluted with 6 M urea. 30 µg of WCE were separated by SDS-PAGE in parallel to 30 µg unbound IP supernatant and 30 µg eluted protein. COUP-TFII-FLAG affinity bead binding is confirmed by decreased FLAG in the Supernatant (flow-thru = unbound proteins) and enriched FLAG in the eluted samples. (PDF) [file pone.0038278.s002.pdf]

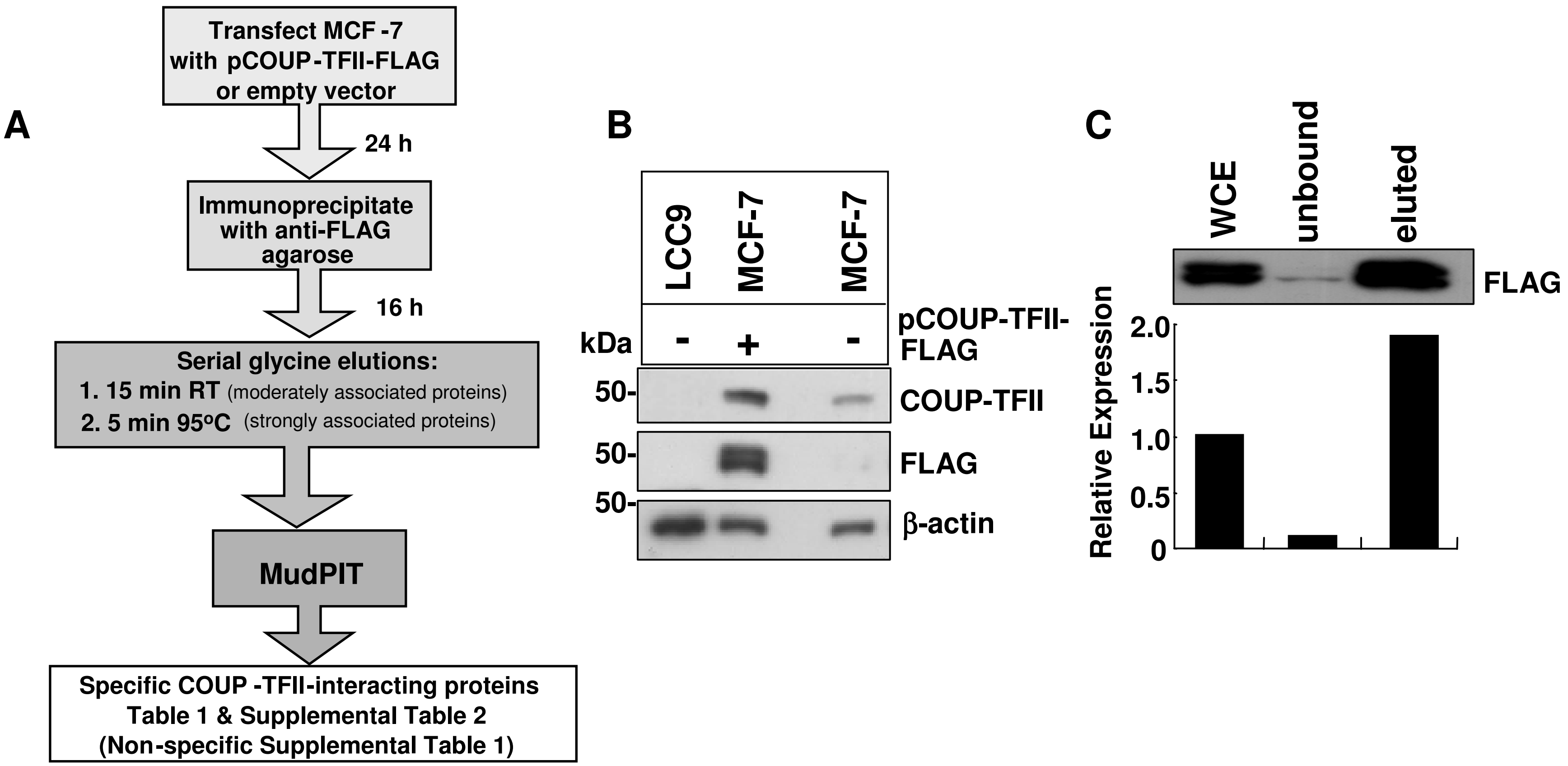

Figure S2: Overexpression of COUP-TFII-FLAG in MCF-7 cells and immunocapture of COUP-TF-FLAG by the anti-FLAG agarose affinity resin. A, Briefly, MCF-7 cells were transiently transfected with C-terminal FLAG-tagged COUP-TFII or empty vector for 24 h as described in Materials and Methods. WCEs were prepared and incubated with EZ view™ Red ANTI-FLAG® M2 Affinity gel (Sigma) for 16 h. After rinsing, proteins were eluted with serial glycine elutions: 1. 15 min at room temperature for proteins associating with immobilized COUP-TFII-FLAG with moderate affinity and 2. 5 min at 95oC to elute proteins bound to the immobilized COUP-TFII-FLAG with high affinity. Immunoprecipitating proteins were analyzed by MudPIT. Non-specific proteins were subtracted from the total interacting proteins to identify proteins specifically interacting with COUP-TFII-FLAG. B, 30 µg of WCE from LCC9, MCF-7 and MCF-7 cells transiently transfected with pCOUP-TFII-FLAG were separated by SDS-PAGE and western blots were performed for COUP-TFII, FLAG and β-actin. Quantitation of the COUP-TFII/β-actin in lanes 2 and 3 indicate a 2-fold increase COUP-TFII in the transfected cells. TAM-R LCC9 cells served as a negative control, as we reported lower COUP-TFII in LCC9 cells compared to parental MCF-7 cells (Riggs *et al* Cancer Res. 66: 10188-98, 2006). Note FLAG signal was only detected in the transfected cells (lane 2), indicating specificity. C, 1 mg of protein in WCE from COUP-TFII-FLAG over-expressing MCF-7 cells was immunocaptured on anti-FLAG agarose affinity beads. COUP-TFII and interacting proteins were eluted with 6 M urea. 30µg of WCE were separated by SDS-PAGE in parallel to 30 µg unbound IP supernatant and 30µg eluted protein. COUP-TFII-FLAG-affinity bead binding is confirmed by decreased FLAG in the Supernatant (flow-thru = unbound proteins) and enriched FLAG in the eluted samples.
