# Supplementary material for: Identification and Characterization of Nucleolin as a COUP-TFII Coactivator of Retinoic Acid Receptor β Transcription in Breast Cancer Cells
Source: PLoS One. 2012 May 31;7(5):e38278. doi: 10.1371/journal.pone.0038278 (PMC3365040; doi:10.1371/journal.pone.0038278)
Supplement: Figure S3 — Testing elution methods for retrieval of COUPTFII-FLAG. ∼1 mg of WCE from MCF-7 cells transfected with pCOUP-TFII-FLAG was immunocaptured on the anti-FLAG affinity resin and eluted with sequential glycine elutions: 1) 10 or 15 min room temperature (lanes 2 and 4 in Experiments A and B, respectively) or 5 min. at 95°C (lanes 3 and 5) in two different experiments: Exp. A or B, as indicated (lanes 2–5). Samples of the indicated eluates were separated by SDS-PAGE and immunoblotted using anti-FLAG antibody. 20 µg of the IP supernatant, containing unbound COUP-TFII-FLAG, was run in parallel as a control (lane 1). As seen in lane 5, incubation with glycine for 15 min. followed by a 5 min. incubation of fresh glycine at 95°C (Exp. B) eluted the most intact COUP-TFII-FLAG (∼50 kDa). The increased temperature and incubation time of glycine in Exp. B resulted in a FLAG-tagged degradation product(s) of ∼20 kDa (lanes 3–5). (PDF) [file pone.0038278.s003.pdf]

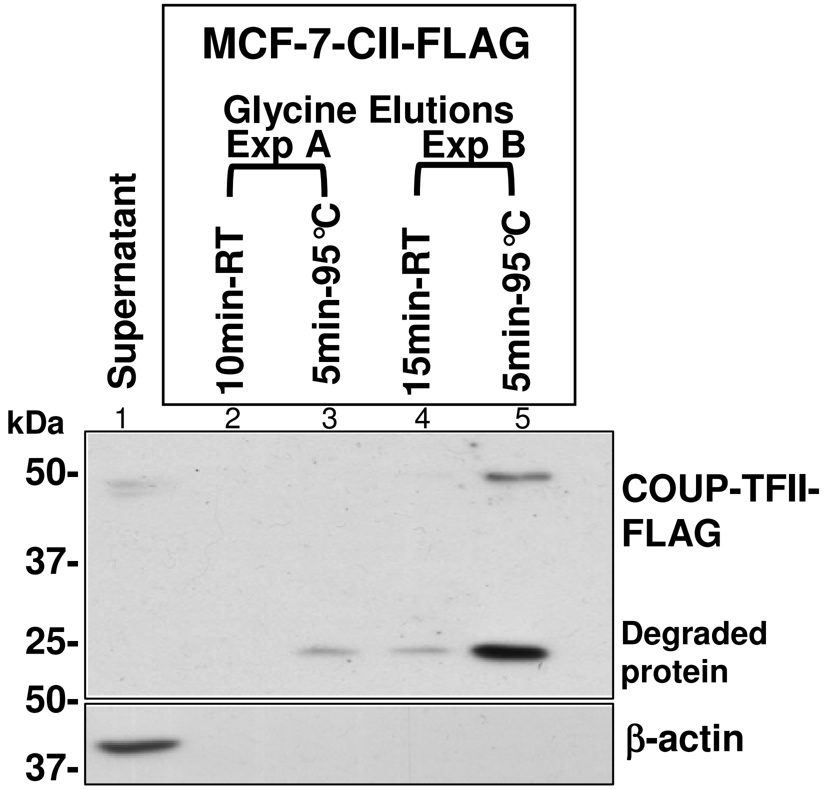

Figure S3: Testing elution methods for retrieval of COUP-TFII-FLAG. ~ 1 mg of WCE from MCF-7 cells transfected with pCOUP-TFII-FLAG was immunocaptured on the anti-FLAG affinity resin and eluted with sequential glycine incubations: 1) 10 or 15 min room temperature (lanes 2 and 4) or 5 min. at 95 °C (lanes 3 and 5) in two different experiments (Exp A or B), as indicated (lanes 2-5). Samples of the indicated eluates were separated by SDS-PAGE and immunoblotted using anti-FLAG antibody. 20 μg of the IP supernatant, containing unbound COUP-TFII-FLAG, was run in parallel as a control (lane 1). As seen in lane 5, incubation with glycine for 15 min. followed by a 5 min. incubation of fresh glycine at 95°C (Exp. B) eluted the most intact COUP-TFII-FLAG (~50 kDa). The increased temperature and incubation time of glycine in Exp. B resulted in a FLAG-tagged degradation product(s) of ~20 kDa (lanes 3-5).
