# Supplementary material for: Identification and Characterization of Nucleolin as a COUP-TFII Coactivator of Retinoic Acid Receptor β Transcription in Breast Cancer Cells
Source: PLoS One. 2012 May 31;7(5):e38278. doi: 10.1371/journal.pone.0038278 (PMC3365040; doi:10.1371/journal.pone.0038278)
Supplement: Figure S4 — Endogenous nuclear nucleolin-COUP-TFII interaction in MCF-7 cells. A, Equal amounts (100 µg) of protein of CE and NE from MCF-7 cells were immunoprecipitated with nucleolin mAB (lanes 3 and 4), mouse (m) IgG (negative control for mAB, lanes 5 and 6), COUP-TFII antisera (lanes 7 and 8), or rabbit (r) IgG (negative control for IPs using COUP-TFII polyclonal antiserum, lanes 9 and 10), followed by western blot for nucleolin and COUPTFII. 20% (20 µg) input NE and CE serve as loading controls (lanes 1 and 2). B, The relative amount of nucleolin and COUP-TFII in the nucleolin IP was plotted relative to expression of each protein in the input (set to 100). COUPTFII in rabbit IgG IPs was not graphed because of the contamination of the heavy IgG chain (lanes 7 and 8, COUP-TFII blot). Western blots demonstrate that: 1) nucleolin interacts with COUP-TFII in the NE of MCF-7 cells (lane 7); 2) nucleolin is not IP'ed with rabbit IgG (lanes 9 and 10); 3) more COUP-TFII interacts with nucleolin in NE IP'ed with nucleolin antibody than with mouse IgG (lane 3 versus lane 5). C, MCF-7 cells were treated with EtOH, 10 nM E2, or 100 nM 4-OHT for 1 h prior to separation of NE and CE. 200 µg of NE or CE were IP'ed with polyclonal COUP-TFII antibody and immunoblotted with a monoclonal antibody (mAB) against nucleolin. The blot was stripped and reprobed with mAB against COUP-TFII. D, 10% input for NE and CE used in IP in part C. (PDF) [file pone.0038278.s004.pdf]

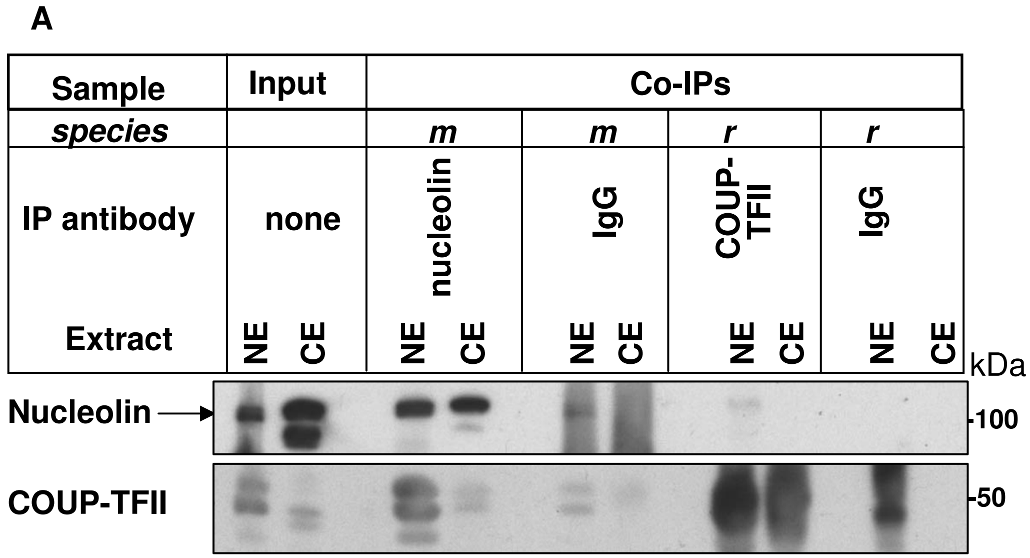

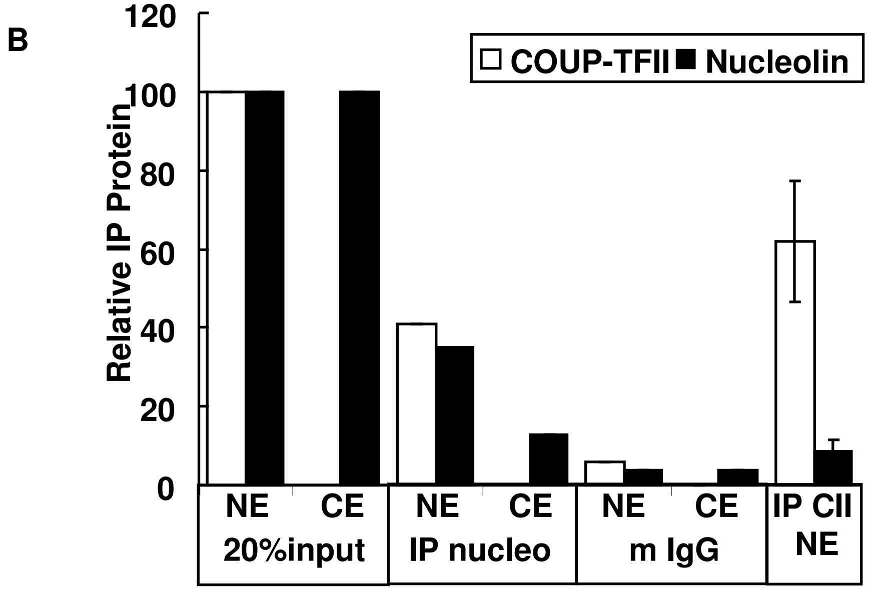

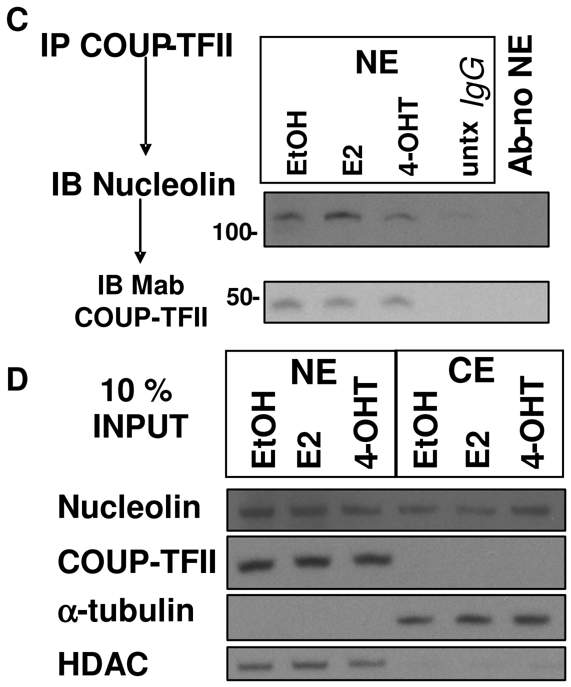

Figure S4: Endogenous nuclear nucleolin-COUP-TFII interaction in MCF-7 cells. A, Equal amounts (100 μg) of protein of CE and NE from MCF-7 cells were immunoprecipitated with nucleolin mAB (lanes 3 and 4), mouse (m) IgG (negative control for mAB, lanes 5 and 6), COUP-TFII antisera (lanes 7 and 8), or rabbit (r) IgG (negative control for IPs using COUP-TFII polyclonal antiserum, lanes 9 and 10), followed by western blot for nucleolin and COUP-TFII. 20% (20 μg) input NE and CE serve as loading controls (lanes 1 and 2). B, The relative amount of nucleolin and COUP-TFII in the nucleolin IP was plotted relative to expression of each protein in the input (set to 100). COUP-TFII in rabbit IgG IPs was not graphed because of the contamination of the heavy IgG chain (lanes 7 and 8, COUP-TFII blot). Western blots demonstrate that: 1) nucleolin interacts with COUP-TFII in the NE of MCF-7 cells (lane 7); 2) nucleolin is not IP'ed with rabbit IgG (lanes 9 and 10); 3) more COUP-TFII interacts with nucleolin in NE IP'ed with nucleolin antibody than with mouse IgG (lane 3 versus lane 5). C, MCF-7 cells were treated with EtOH, 10 nM E2, or 100 nM 4-OHT for 1 h prior to separation of NE and CE. 200 μg of NE or CE were IP'ed with polyclonal COUP-TFII antibody and immunoblotted with a monoclonal antibody (mAB) against nucleolin. The blot was stripped and re-probed with mAB against COUP-TFII. D, 10% input for NE and CE used in IP in part C.
