# Supplementary material for: Identification and Characterization of Nucleolin as a COUP-TFII Coactivator of Retinoic Acid Receptor β Transcription in Breast Cancer Cells
Source: PLoS One. 2012 May 31;7(5):e38278. doi: 10.1371/journal.pone.0038278 (PMC3365040; doi:10.1371/journal.pone.0038278)
Supplement: Figure S5 — ChIP of COUP-TFII-FLAG on the RARB2 promoter in MCF-7 cells. A, Chromatin immunoprecipitation was performed in MCF-7 cells transfected with pIRES-COUP-TFII-FLAG or empty vector, serum starved for 48 h, and treated with 1 µM atRA for 1 h. Following Q-PCR using primers to the RARB2 promoter as described in Materials and Methods, duplicate samples were run on a 2% agarose gel. B, Only EV – EtOH set to 1. atRA increased COUP-TFIIFLAG binding to the RARB2 promoter 32%. Significantly different p<0.05: * to EV – EtOH, ** to EV – atRA, ‡ to CII – EtOH. (PDF) [file pone.0038278.s005.pdf]

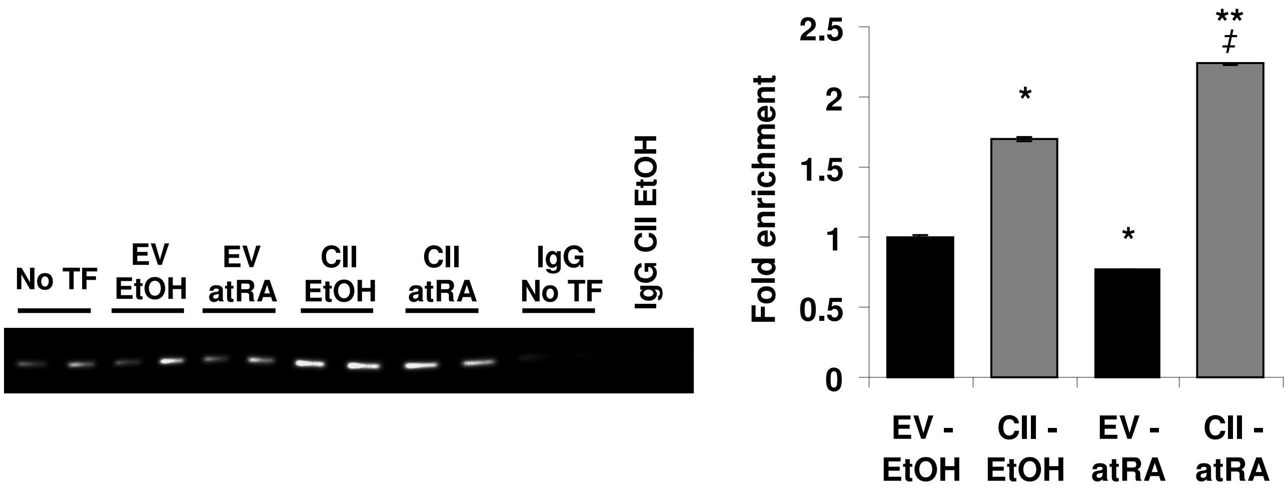

Figure S5: ChIP of COUP-TFII-FLAG on the *RARB2* promoter in MCF-7 cells. A, Chromatin immunoprecipitation was performed in MCF-7 cells transfected with pIRES-COUP-TFII-FLAG or empty vector, serum starved for 48 h, and treated with 1 μM atRA for 1 h. Following Q-PCR using primers to the *RARB2* promoter as described in Materials and Methods, duplicate samples were run on a 2% agarose gel. B, Only EV – EtOH set to 1. atRA increased COUP-TFII-FLAG binding to the RARB2 promoter 32%. Signific. different $p < 0.05$: * to EV – EtOH, ** to EV – atRA, ‡ to CII – EtOH.
