# Supplementary material for: Identification and Characterization of Nucleolin as a COUP-TFII Coactivator of Retinoic Acid Receptor β Transcription in Breast Cancer Cells
Source: PLoS One. 2012 May 31;7(5):e38278. doi: 10.1371/journal.pone.0038278 (PMC3365040; doi:10.1371/journal.pone.0038278)
Supplement: Figure S6 — Expression of COUP-TFII and nucleolin in T47D and MCF-7 cells. A, WCE (50 µg) were Western blotted for COUP-TFII and nucleolin expression. The blot was stripped and re-probed for β-actin. B, The ratio of nucleolin/β-actin and COUP-TFII/β-actin for each cell lines was plotted. These data are the average of 3 separate experiments. The lower COUP-TFII expression in T47D agrees with the higher CT values for NR2F2 in T47D. (PDF) [file pone.0038278.s006.pdf]

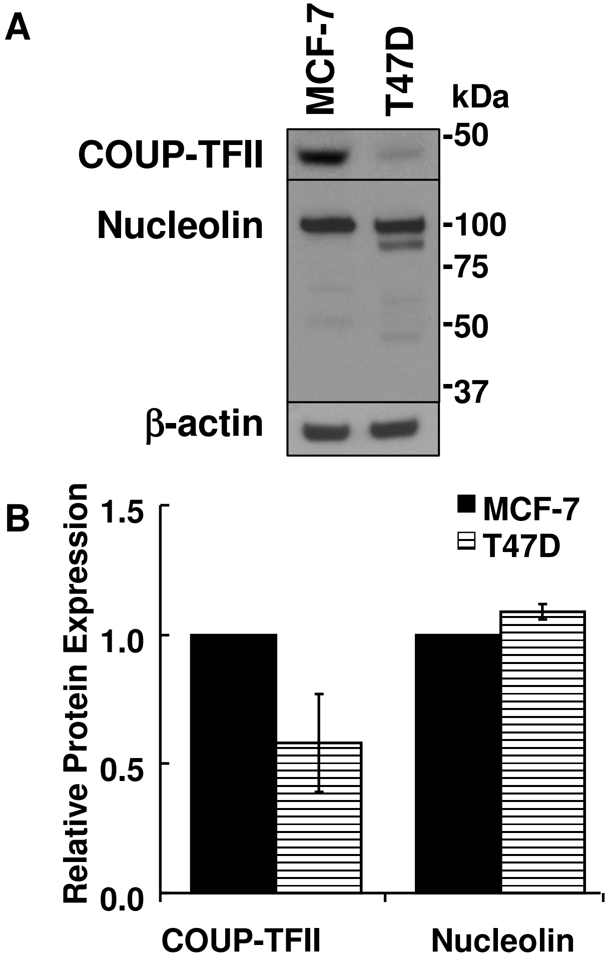

Figure S6: Expression of COUP-TFII and nucleolin in T47D and MCF-7 cells. A, WCE (50 µg) were Western blotted for COUP-TFII and nucleolin expression. The blot was stripped and re-probed for β-actin. B, The ratio of nucleolin/β-actin and COUP-TFII/β-actin for each cell lines was plotted. These data are the average of 3 separate experiments. The lower COUP-TFII expression in T47D agrees with the higher CT values for *NR2F2* in T47D.
