# Supplementary material for: Identification and Characterization of Nucleolin as a COUP-TFII Coactivator of Retinoic Acid Receptor β Transcription in Breast Cancer Cells
Source: PLoS One. 2012 May 31;7(5):e38278. doi: 10.1371/journal.pone.0038278 (PMC3365040; doi:10.1371/journal.pone.0038278)
Supplement: Figure S7 — Effect of cell treatments on NR2F2 (COUP-TFII) expression in MCF-7 cells. A, Schematic diagram of transfection and treatment of MCF-7 cells. MCF-7 cells were transfected with equal amounts of pTAG2 control vector or pCMV-nucleolin for 24 h., treated with 10 µM CRO or AS1411, as indicted for 24 h, and 1 µM atRA was added for the last 6 h. RNA was harvested and Q-PCR performed. NR2F2 (B) values were normalized to GAPDH. Values are the average of 6 separate experiments ± SEM. * significantly different, p<0.05 in one way ANOVA followed by Bonferroni multiple comparison test. Note that there was no statistical difference between the pTAG+AS1411 versus Nucl+AS1411 or AS1411 sample measurements of NR2F2. (PDF) [file pone.0038278.s007.pdf]

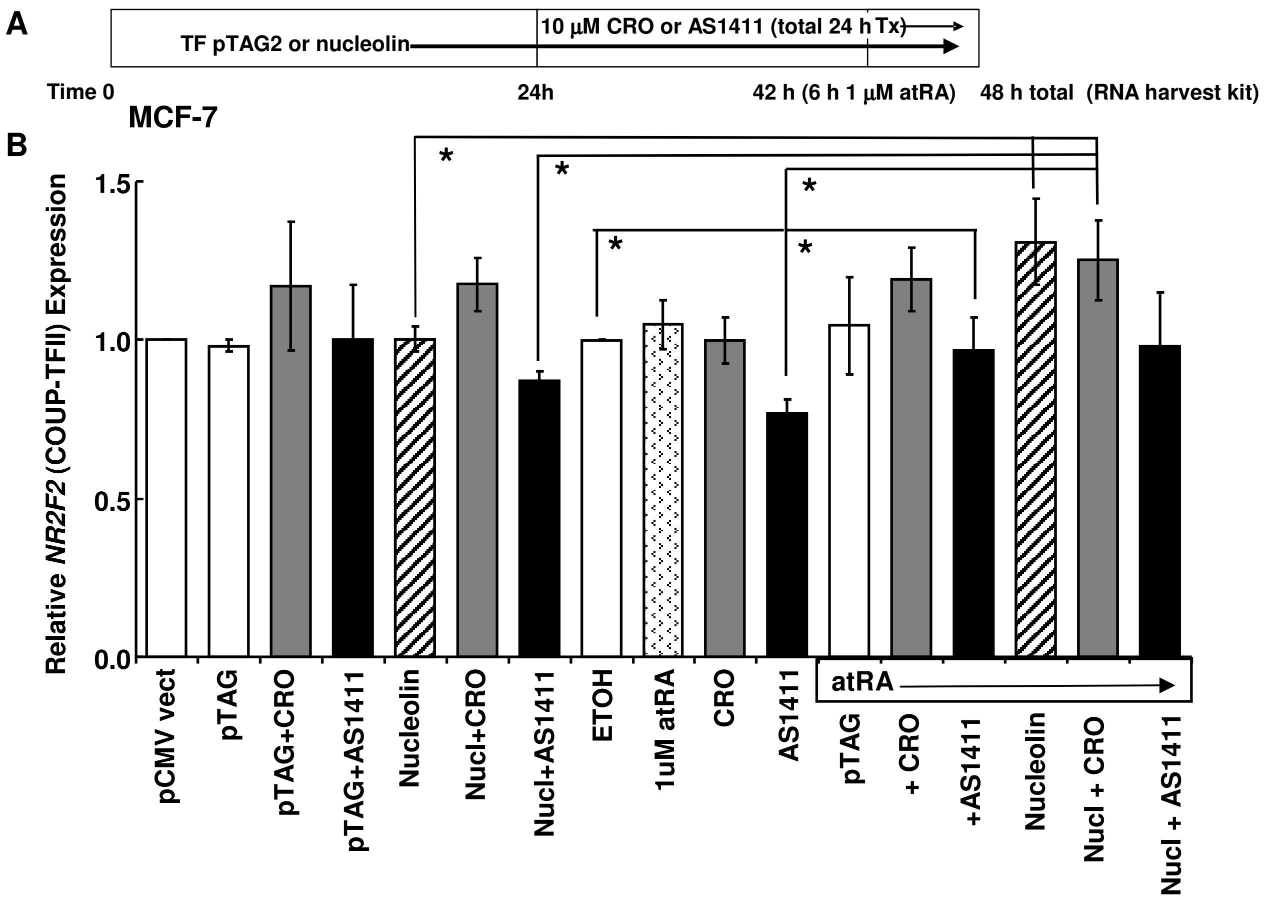

Figure S7: Effect of cell treatments on *NR2F2* (COUP-TFII) expression in MCF-7 cells. A, Schematic diagram of transfection and treatment of MCF-7 cells. MCF-7 cells were transfected with equal amounts of pTAG2 control vector or pCMV-nucleolin for 24 h., treated with 10 μM CRO or AS1411, as indicted for 24 h, and 1 μM atRA was added for the last 6 h. RNA was harvested and Q-PCR performed. B, *NR2F2* values were normalized to GAPDH. Values are the average of 6 separate experiments ± SEM. * signific. different, $p < 0.05$ in one way ANOVA followed by Bonferroni multiple comparison test. Note that there was no statistical difference between the pTAG+AS1411 *versus* Nucl+AS1411 or AS1411 sample measurements of *NR2F2.*
