# Supplementary material for: Identification and Characterization of Nucleolin as a COUP-TFII Coactivator of Retinoic Acid Receptor β Transcription in Breast Cancer Cells
Source: PLoS One. 2012 May 31;7(5):e38278. doi: 10.1371/journal.pone.0038278 (PMC3365040; doi:10.1371/journal.pone.0038278)
Supplement: Figure S8 — Neither AS1411 nor CRO inhibit MCF-7 cell viability after 4 d. MCF-7 cells were treated with the indicated concentrations of AS1411 or CRO and cell viability was measured by an MTT assay (A490 nm, Promega CellTitre assay). Values are the average of 4 determinations ± SEM. (PDF) [file pone.0038278.s008.pdf]

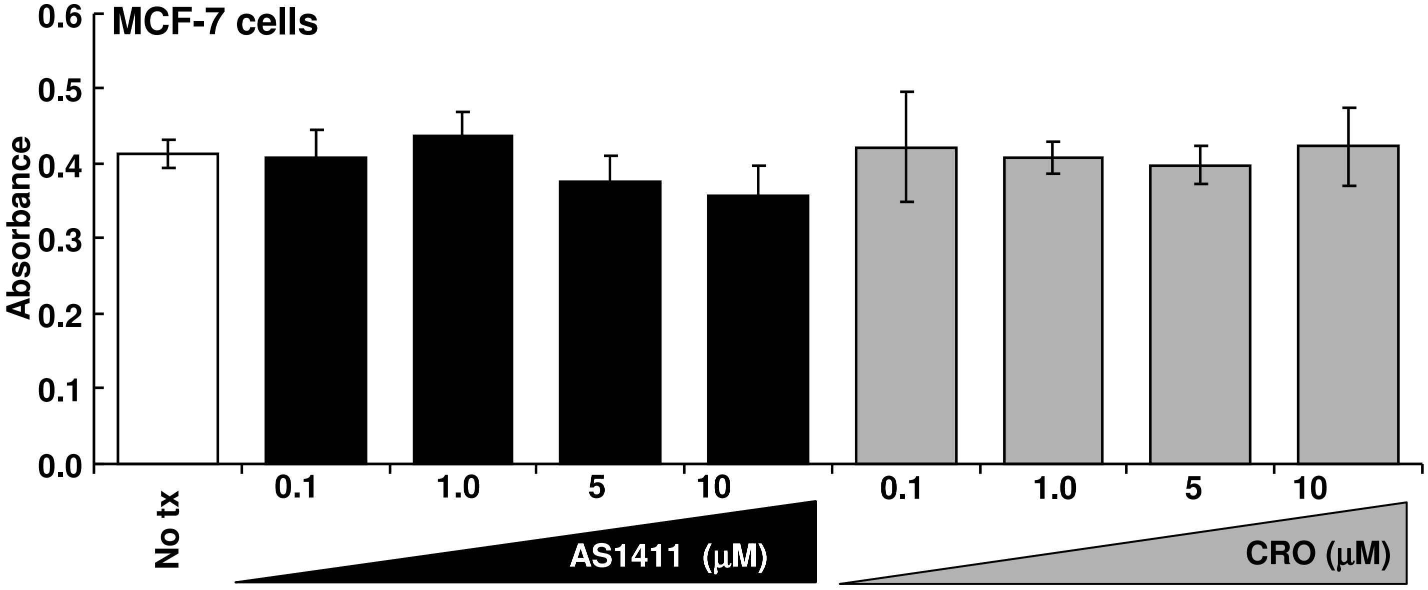

Figure S8: Neither AS1411 nor CRO inhibit MCF-7 cell viability after 4 d. MCF-7 cells were treated with the indicated concentrations of AS1411 or CRO and cell viability was measured by an MTT assay (A490nm, Promega CellTitre assay). Values are the average of 4 determinations ± SEM.
