# Supplementary material for: Identification and Characterization of Nucleolin as a COUP-TFII Coactivator of Retinoic Acid Receptor β Transcription in Breast Cancer Cells
Source: PLoS One. 2012 May 31;7(5):e38278. doi: 10.1371/journal.pone.0038278 (PMC3365040; doi:10.1371/journal.pone.0038278)
Supplement: Figure S9 — Oncomine examination of NR2F2 expression in breast tumors. Data mining in Oncomine microarray data sets for NR2F2 in breast cancer identified that: A, NR2F2 expression is significantly higher in ERα+ breast tumors (p<0.007). B. NR2F2 is significantly lower in metastatic breast tumors (p<0.05). Data are from (van de Vijver MJ, He YD, van't Veer LJ, et al. A gene-expression signature as a predictor of survival in breast cancer. N Engl J Med 2002; 347: 1999–2009). (PDF) [file pone.0038278.s009.pdf]

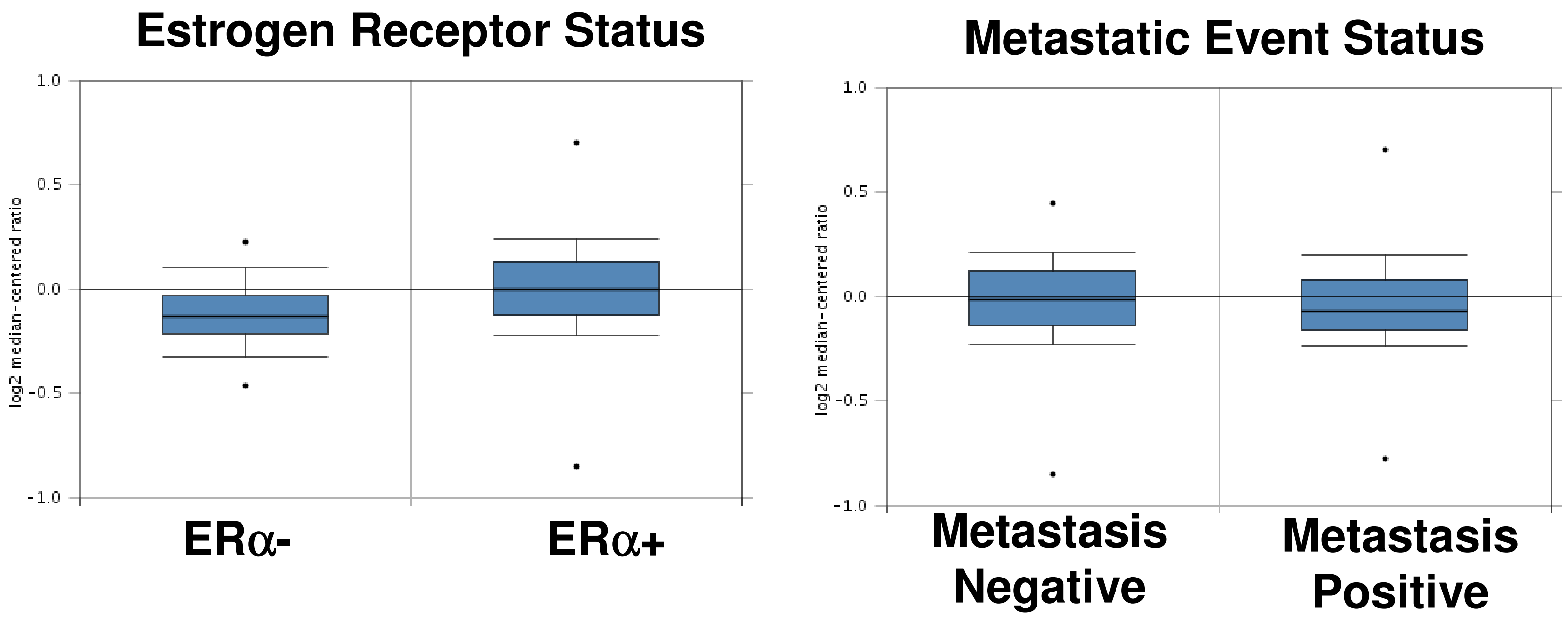


**Figure S9: Oncomine examination of *NR2F2* expression in breast tumors**. Data mining in Oncomine microarray data sets for NR2F2 in breast cancer identified that: A, *NR2F2* expression is significantly higher in ERα+ breast tumors ($p < 0.007$). B. *NR2F2* is significantly lower in metastatic breast tumors ($p < 0.05$). Data are from (van de Vijver MJ, He YD, van't Veer LJ, et al. A gene-expression signature as a predictor of survival in breast cancer. N Engl J Med 2002; 347: 1999-2009).
