# Supplementary material for: Identification and Characterization of Nucleolin as a COUP-TFII Coactivator of Retinoic Acid Receptor β Transcription in Breast Cancer Cells
Source: PLoS One. 2012 May 31;7(5):e38278. doi: 10.1371/journal.pone.0038278 (PMC3365040; doi:10.1371/journal.pone.0038278)
Supplement: Table S1 — Identification of proteins in MCF-7 WCE that non-specifically (NS) interact with the anti-FLAG-affinity resin. 5 mg protein in WCE from EMPTY-FLAG vector-transfected MCF-7 cells was incubated with anti-FLAG affinity gel (right side of Supplemental Figure 1), eluted with 0.1 M glycine, pH 3.5 for 15 min. at RT, and subjected to MudPIT peptide identification. Matched number (No) indicates the number of sequenced peptides that match the full length protein. Coverage indicates the % of the total protein matched. (DOC) [file pone.0038278.s010.doc]

**Table S1: Identification of proteins in MCF-7 WCE that non-specifically (NS) interact with the anti-FLAG-affinity resin.**

| **Protein name** | **Accession** | **Mass** | **pl** | **Matched** | **Coverage** |
| --- | --- | --- | --- | --- | --- |
|  | **(GI)** | **(Mr)** |  | **(No)** | **(%)** |
| **Cytoskeletal proteins** |  |  |  |  |  |
| Actin cytoplasmic 1 | P60709 | 41710 | 5.4 | 4 | 15.5 |
| **Tropomyosin alpha-4 chain** | P67936 | 28505 | 4.7 | 4 | 17.3 |
| Tropomyosin alpha-3 chain | P06753 | 29015 | 4.8 | 4 | 18.1 |
| Myosin-9 | P35579 | 226392 | 4.6 | 4 | 3.0 |
| **Tropomyosin beta chain** | P07951 | 32970 | 4.7 | 5 | 23.6 |
| Tropomyosin alpha-1 chain | P09493 | 32689 | 4.7 | 4 | 14.8 |
| Myosin light polypeptide 6 | P60660 | 16788 | 4.6 | 3 | 22.7 |
|  |  |  |  |  |  |
| **Histones** |  |  |  |  |  |
| Histone H4 | P62805 | 11229 | 11.4 | 3 | 29.4 |

5 mg protein in WCE from EMPTY-FLAG vector-transfected MCF-7 cells was incubated with anti-FLAG affinity gel (right side of Supplemental Figure 1), eluted with 0.1 M glycine, pH 3.5 for 15 min. at RT, and subjected to MudPIT peptide identification. Matched number (No) indicates the number of sequenced peptides that match the full length protein. Coverage indicates the % of the total protein matched.
