# Supplementary material for: Identification and Characterization of Nucleolin as a COUP-TFII Coactivator of Retinoic Acid Receptor β Transcription in Breast Cancer Cells
Source: PLoS One. 2012 May 31;7(5):e38278. doi: 10.1371/journal.pone.0038278 (PMC3365040; doi:10.1371/journal.pone.0038278)
Supplement: Table S2 — Identification of proteins ‘moderately’ associated with COUP-TFII in MCF-7 cells. 5 mg protein in WCE from pCOUP-TFII-FLAG transfected, EtOH-treated MCF-7 cells was incubated with anti-FLAG affinity gel (left side of Supplemental Figure 1), eluted with 0.1 M glycine, pH 3.5 for 15 h at RT, and subjected to MudPIT peptide identification. Matched number (No) indicates the number of sequenced peptides that match the full length protein. Coverage indicates the % of the total protein matched. This table excludes proteins that were nonspecifically associated with the anti-FLAG affinity gel as summarized in Supplemental Tables 1. (DOC) [file pone.0038278.s011.doc]

**Table S2: Identification of proteins ‘moderately’ associated with COUP-TFII in MCF-7 cells.**

| **Protein name *(gene*)** | **Accession** | **Mass** | **pl** | **Matched** | **Coverage** |
| --- | --- | --- | --- | --- | --- |
|  | **(GI)** | **(Mr)** |  | **(No)** | **(%)** |
| **Ribonucleoproteins** |  |  |  |  |  |
| Heterogeneous nuclear ribonucleoprotein D0 | Q14103 | 22435 | 11.3 | 2 | 10.2 |
|  |  |  |  |  |  |
| **Histones** |  |  |  |  |  |
| Histone H2A.Z | P0C0S5 | 13414 | 10.9 | 2 | 7.9-16 |
| Core histone macro-H2A.1 | O75367 | 39445 | 10.1 | 2 | 5.4 |
| Histone H1.5 | P16401 | 22435 | 11.3 | 3 | 10.2 |
| Histone H1.2 | P16403 | 21221 | 11.3 | 3 | 10.8 |
| Histone H3.1 | P68431 | 15263 | 11.3 | 2 | 10.4 |
|  |  |  |  |  |  |
| **Other Proteins** |  |  |  |  |  |
| Heat Shock 70 kDa protein B (*HSPA7*) | P48741 | 26890 | 7.6 | 3 | 16.2 |
| Heat Shock 70 kDa protein 1 HSP70 (*HSPA1A*) | P08107 | 70009 | 5.6 | 3 | 6.2 |
| Chromobox Protein Homolog 3 (*CBX3*) | Q13185 | 19708 | 5.1 | 3 | 6.4 |

5 mg protein in WCE from pCOUP-TFII-FLAG transfected, EtOH-treated MCF-7 cells was incubated with anti-FLAG affinity gel (left side of Supplemental Figure 1), eluted with 0.1 M glycine, pH 3.5 for 15 h at RT, and subjected to MudPIT peptide identification. Matched number (No) indicates the number of sequenced peptides that match the full length protein. Coverage indicates the % of the total protein matched. This table excludes proteins that were nonspecifically associated with the anti-FLAG affinity gel as summarized in Supplemental Tables 1.
