# Supplementary material for: Identification and Characterization of Nucleolin as a COUP-TFII Coactivator of Retinoic Acid Receptor β Transcription in Breast Cancer Cells
Source: PLoS One. 2012 May 31;7(5):e38278. doi: 10.1371/journal.pone.0038278 (PMC3365040; doi:10.1371/journal.pone.0038278)
Supplement: Methods S1 — (DOC) [file pone.0038278.s012.doc]

Methods S1:

**Transient transfection with pCOUP-TFII-FLAG.**  MCF-7 cells, purchased from ATCC, and used at passages <10, were transiently transfected with 2 μg pCOUP-TFII-FLAG using the P-20 program of the Amaxa Nucleofection System. Cells were ‘serum-starved’ in phenol red-free IMEM supplemented with 5% dextran coated charcoal stripped FBS (DCC-FBS) for 24 h before treatment with vehicle control ethanol (EtOH) for 2 h as indicated in the experimental design (Fig. 1).

**Protein identification by multidimensional protein identification technology (MudPIT).** Proteins eluted from the FLAG-affinity gel (Fig. 1) were digested by trypsin, desalted with C18 spin columns, dried by speedvac, and fractionated by strong cation exchange (SCX). The desalted samples were dissolved with loading buffer containing 20% acetonitrile (ACN), 100 mM acetic acid (HAc), and 2 mM NH_4_Acetate and then loaded on a pre-cleaned SCX cartridge from Michrom Bioresources. After washing twice, peptides were eluted stepwise with 20 μl of HAc/NH_4_Ac buffers with increasing ionic strength and pH (15 buffers and up to 10 mM HAc/615 mM NH_4_Ac, pH 6.5). The SCX fractions were concentrated by speedvac and diluted to 7 μl with 5% ACN/0.1% formic acid. 5 μl of the samples were loaded to CapLC capillary LC system from Waters and peptides in the samples were separated with a 3.5 μm Symmetry C18 column (75 μm × 150 mm, Waters). MS/MS spectra of the peptides were acquired by Q-TOF mass spectrometer (Waters) in data dependent mode. Proteins were identified by comparing MS/MS spectra with sequences in Swiss-Prot database by ProteinLynx from Waters.

**[Immuno](http://www.sciencedirect.com/science?_ob=ArticleURL&_udi=B6T20-4GDK5R4-2&_coverDate=06%2F16%2F2005&_alid=294868305&_rdoc=1&_fmt=&_orig=search&_qd=1&_cdi=4904&_sort=d&view=c&_acct=C000011238&_version=1&_urlVersion=0&_userid=134779&md5=dfc63173e8abdd9fe05d0f27a595930f#SECX5%23SECX5)fluorescence staining of pCOUP-TFII-FLAG.** Cells were grown on coverslips, washed with PBS, fixed with cold methanol/acetone 1:1 for 5 min, and washed twice with cold PBS. After blocking with 1% goat serum and 0.3 % Triton X-100 in PBS for 30 min, primary FLAG antibody (Sigma) was added (1: 300 dilution) for 1 h. After removing the primary antibody, the cells were stained with secondary anti-mouse antibody labeled with Zenon^TM^ Alexa Fluor 488 (Molecular Probes). Cells were then incubated with ProLong® Gold antifade reagent with DAPI (Molecular Probes). Images (Fig. S1) were captured using a Zeiss Axiovert200 inverted microscope with a 63x objective lens using AxioVision Release 4.3 software.
